# Enantioselective Iron/Bisquinolyldiamine Ligand-Catalyzed Oxidative Coupling Reaction of 2-Naphthols

**DOI:** 10.3390/molecules25040852

**Published:** 2020-02-14

**Authors:** Lin-Yang Wu, Muhammad Usman, Wen-Bo Liu

**Affiliations:** Sauvage Center for Molecular Sciences; Engineering Research Center of Organosilicon Compounds & Materials, Ministry of Education; College of Chemistry and Molecular Sciences; Wuhan University, Wuhan 430072, Hubei, China; 2016282030199@whu.edu.cn (L.-Y.W.); whitestar399@hotmail.com (M.U.)

**Keywords:** iron catalysis, asymmetric catalysis, nitrogen ligand, oxidative coupling, BINOL synthesis

## Abstract

An iron-catalyzed asymmetric oxidative homo-coupling of 2-naphthols for the synthesis of 1,1′-Bi-2-naphthol (BINOL) derivatives is reported. The coupling reaction provides enantioenriched BINOLs in good yields (up to 99%) and moderate enantioselectivities (up to 81:19 er) using an iron-complex generated in situ from Fe(ClO_4_)_2_ and a bisquinolyldiamine ligand [(1*R*,2*R*)-*N*^1^,*N*^2^-di(quinolin-8-yl)cyclohexane-1,2-diamine, **L1**]. A number of ligands (**L2**–**L8**) and the analogs of **L1**, with various substituents and chiral backbones, were synthesized and examined in the oxidative coupling reactions.

## 1. Introduction

**Figure 1:**
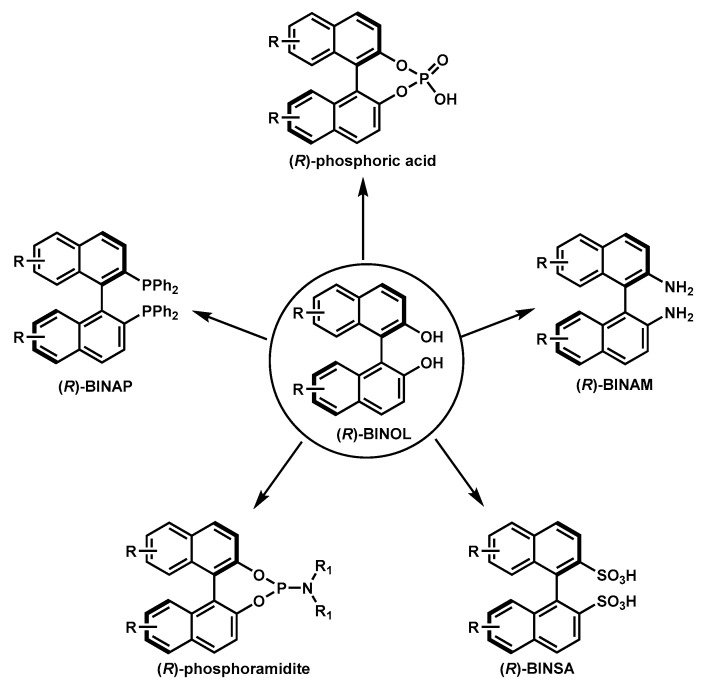
Representative 1,1′-Bi-2-naphthol (BINOL)-derived ligands/catalysts.

Axially chiral compounds (atropisomers) have aroused much attention from organic chemists due to their prevalence in natural products, bioactive molecules, functional materials, and their wide applications in asymmetric transformations [1]. Many elegant methods have been established for the asymmetric synthesis of axially chiral compounds, both employing transition-metal catalysts [2] and organocatalysts [1]. In particular, 1,1′-Bi-2-naphthol (BINOL) is one of the most useful structural motifs and ligand substructures in asymmetric catalysis [3,4,5,6,7,8,9]. Since the pioneering report by the Noyori group utilizing enantioenriched BINOL as the ligand in asymmetric catalysis [10], numerous BINOL-derived ligands/catalysts (i.e., BINAP [11], BINAM [12], chiral phosphoric acid [13], chiral phosphoramidite [14], and BINSA [15]; Figure 1) have been designed and synthesized. The emergence of such a library of ligands/catalysts has brought marvelous contributions to the synthetic community, with tremendously efficient asymmetric transformations such as reductive coupling [16], allylation [17], ene-type [18], and Aldol [19] reactions and axial chirality assembly [20].

**Figure 2:**
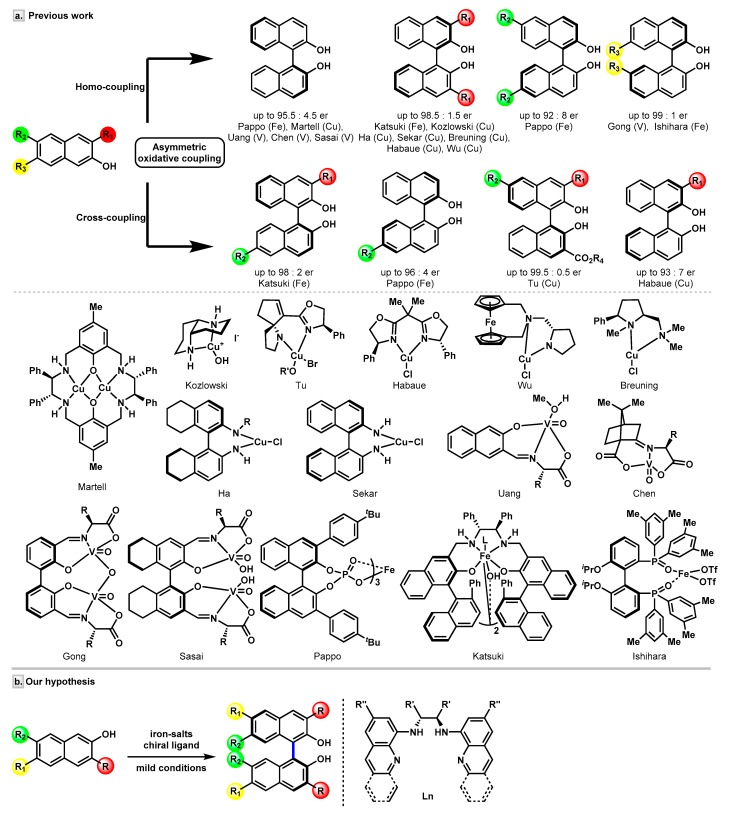
Transition metal-catalyzed coupling for the enantioselective synthesis of BINOLs. (**a**) The previous examples of homo- or cross-coupling of 2-naphthols. (**b**) Our proposed asymmetric oxidative homo-coupling of 2-naphthols using iron/bisquinolyldiamine catalyst.

In the past few decades, enormous efforts have been devoted to the enantioselective assembly of BINOL scaffolds. Transition-metal-catalyzed asymmetric oxidative coupling of 2-naphthols have shown its power in the synthesis of BINOLs (Figure 2a). Efficient vanadium-catalyzed [21] protocols were reported by the Uang [22], Chen [23], Gong [24,25,26], and Sasai [27,28,29] groups, independently. Many research groups, including Nakajima, Kozlowski, and others, have successfully developed a series of copper-catalyzed coupling reactions of 2-naphthols [30,31,32,33,34,35,36,37,38,39,40,41,42,43,44]. Recently, a notable work by the Tu group [45] established a Cu/SPDO (spirocyclic pyrrolidine oxazoline) complex-catalyzed cross-coupling reaction to synthesize 3,3′-disubstituted BINOLs. Nevertheless, iron-catalyzed coupling strategies in this area have not been explored so far. Only a handful of remarkable iron catalysts, namely, an iron-salen complex reported by Katsuki and co-workers [46,47], a chiral diphosphine oxide–iron(II) complex developed by the Ishihara group [48], and an iron-chiral phosphoric acid (CPA) catalyst introduced by the Pappo group [49,50], have been disclosed to date (Figure 2a).

Recently, we have developed an iron-catalyzed direct amination of aliphatic C-H bonds [51,52], and it was interesting to find that the catalysts used were simply generated by in situ mixing of an iron salt and an aminopyridine ligand. Inspired by these results, we envisaged that introducing a chiral aminopyridine-type ligand might impart chirality to the products. Our attention was drawn to *N,N′*-dimethyl-*N*,*N′*-bis(8-quinolyl)-cyclohexanediamine (BQCN), developed by the Che group and successfully applied in iron-catalyzed asymmetric *cis*-dihydroxylation of alkenes [53] and, most recently, in Friedel-Crafts reactions [54]. Since our previous studies revealed that free secondary amine ligand presented a good reactivity [51], we were wondering whether the *N*-unprotected bis(8-quinolyl)-cyclohexanediamine ligand (bisquinolyldiamine, **L1**), which is synthesized straightforwardly from 8-haloquinoline and diamine, is capable of controlling the selectivities in the iron-catalyzed oxidative coupling of 2-naphthols. Herein, we report the studies toward the synthesis of the amino ligands and their applications in the synthesis of optically active BINOL derivatives via an oxidative homo-coupling reaction of 2-naphthols under mild conditions (Figure 2b).

## 2. Results and Discussion

### 2.1. Synthesis of Bisquinolyldiamine Ligands

**Figure 3:**
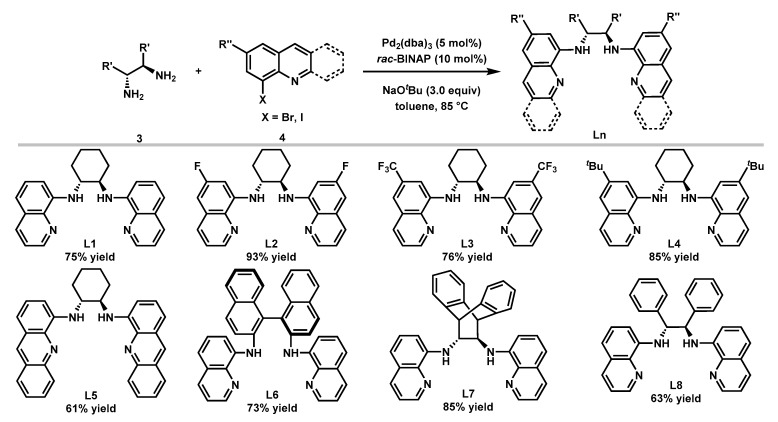
Ligands synthesis.

The Buchwald-Hartwig C–N coupling reaction [55,56] was used for the synthesis of a variety of bisquinolyldiamine ligands following the literature procedure [53]. As shown in Figure 3, with the catalyst derived from 5 mol% Pd_2_(dba)_3_ and 10 mol% *rac*-BINAP, eight ligands were synthesized in moderate to good yields. The chiral backbones of these ligands include (1*R*,2*R*)-cyclohexane-1,2-diamine (**L1**), (*R*)-[1,1′-binaphthalene]-2,2′-diamine (**L6**), (12*R*)-9,10-dihydro-9,10-ethanoanthracene-11,12-diamine (**L7**), and (1*R*,2*R*)-1,2-diphenylethane-1,2-diamine (**L8**). Ligands with a variety of electronically differentiated substituents at the C6 position of the quinoline moiety (**L2–L4**) and an acridine-derived ligand (**L5**) were also prepared to probe the electronic and steric effects of the ligands on the reaction.

### 2.2. Reaction Investigation

To test our hypothesis, we selected 2-naphthol (**1a**) as the model substrate for optimization of reaction conditions (Table 1). All reactions were carried out with the catalyst generated in situ by stirring Fe(ClO_4_)_2_ and **L1** for 30 min before the addition of the substrate, and under atmospheric dioxygen. Various solvents (methanol, dichloroethane, chloroform, toluene, and chlorobenzene) were screened first (entries 1–5) and resulted in moderate conversions and enantioselectivities, except toluene and methanol. The reaction in chlorobenzene gave a better balance between reactivity and selectivity (53% conv. and 79:21 er, entry 5). The addition of 4Å MS led to a slightly higher enantioselectivity with partial conversion in a much shorter reaction time (entries 5 vs. 6). We were delighted to find that increasing the temperature from 30 °C to 50 °C delivered 39% conversion with comparable enantioselectivity (entry 7). Further increase in temperature (i.e., 70 °C and 90 °C) resulted in lower enantioselectivities (entries 8 and 9). Then, the reaction with different iron salts were investigated, including Fe(ClO_4_)_3_, Fe(OAc)_2_, Fe(OTf)_2_, Fe(acac)_2_, and FeCl_2_, but failed to provide better results (entries 10–14). The ratio of iron precursor versus **L1** was also examined (entries 14–19). Surprisingly, increasing Fe(ClO_4_)_2_-loading from 5 mol% to 10 mol% improves the efficiency without affecting the enantioselectivity and delivered the coupling product **2a** in 84% isolated yield with 80:20 er (entries 15 and 16). Although further increasing Fe(ClO_4_)_2_ to 12.5 mol% improved the yield, a slightly diminished er was also observed (entry 17). Finally, reactions with the catalysts derived from the diamine ligand (**L2**–**L8**) were inspected. Electron-withdrawing groups-substituted ligands (**L2** and **L3**) showed excellent reactivities but with low enantioselectivities (entries 20 and 21). In contrast, ligand **L4** with an electron-donating substituent delivered the product in reduced yield, albeit with good er (entry 22). Upon further screening, the ligands (**L5**–**L8**) bearing different chiral backbones, lower yield, and er were obtained with **L5**, while ligands **L6**–**L8** failed to give any product (entries 23–26).

### 2.3. Substrates Scope

**Scheme 1:**
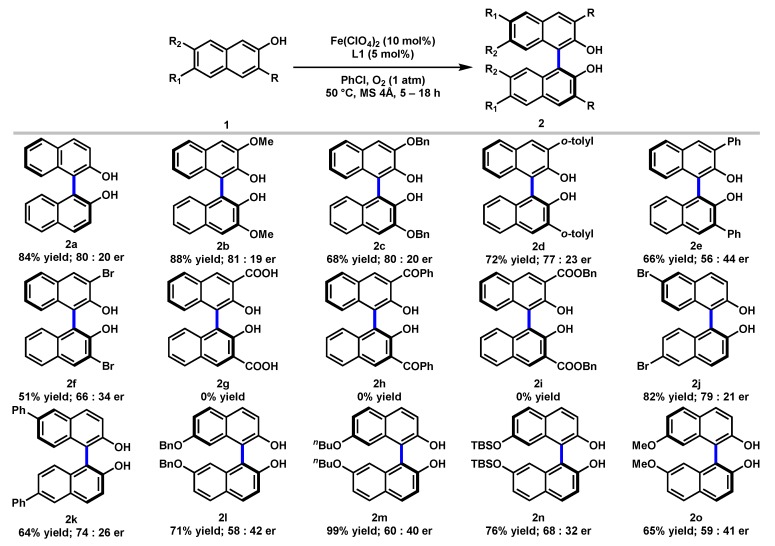
Substrates Scope *^a^. ^a^* Reactions conducted with Fe(ClO_4_)_2_ (10 mol%), **L1** (5 mol%), substrate **1** (0.5 mmol), and 4Å MS (150 mg) in PhCl (5 mL) under oxygen atmosphere (1 atm) at 50 °C. Percentage represented isolated yields. Er determined by HPLC.

Next, we investigated the scope of the iron-catalyzed asymmetric oxidative coupling reaction, and the results were summarized in Scheme 1. A variety of substituted 2-naphthols with electronic and steric properties were examined. Substrate **1** containing functionalities at the C3-position, including OMe, OBn, *o*-tolyl, and Ph were successfully converted into the coupling products (**2b**–**2e**) in 56–88% yields with 56:44 to 81:19 er. The substrate bearing an electron-withdrawing Br substitution at the C3-position was also converted into the corresponding product (**2f**) in 51% yield with 79:21 er. The electronic effects of different functionalities are clearly demonstrated by the observation that electron-donating groups delivered higher yield (e.g., C3-OMe 88%, **2b** vs. C3-Br 51%, **2f**). However, C3-substituted substrates with carbonyl functionalities like CO_2_H, COPh, and CO_2_Bn failed to give any products (**2g**–**2i**). When C6-Br-substituted 2-naphthol was applied, the desired product (**2j**) was obtained in 82% yield with 79:21 er. A C6-phenyl-substituted 2-naphthol also resulted in 64% yield and 74:26 er (**2k**). Moreover, C7-substituted (BnO, *^n^*BuO, TBSO, and MeO) substrates were also effectively coupled and delivered the corresponding products (**2l**–**2o**) in 65–99% yields, albeit with dramatically diminished er.

## 3. Materials and Methods

### 3.1. General Information

Unless otherwise noted, all reagents were purchased commercially and used without further purification. Petroleum ether (PE) (60–90 °C), ethyl acetate (EA), and dichloromethane (DCM) were used for silica gel chromatography. MeCN, toluene, DMF, and THF were purchased commercially or were dried by passage through an activated alumina column under argon [57]. PhCl, CHCl_3_, MeOH, and acetone were freshly distilled after drying over CaH_2_. ^1^H-NMR spectra were recorded at room temperature on a Bruker ADVANCE III 400 MHz spectrometer and were reported relative to residual Chloroform-*d* (δ 7.26 ppm) or DMSO-d*6* (δ 2.50 ppm). ^13^C-NMR spectra were recorded on a Bruker ADVANCE III 400 MHz spectrometer (100 MHz) and were reported relative to Chloroform-*d* (δ 77.16 ppm). ^19^F-NMR spectra were recorded on a Bruker ADVANCE III 400 MHz spectrometer (376 MHz). Data for ^1^H-NMR were reported as chemical shift (δ ppm) (multiplicity, coupling constant (Hz), and integration) using standard abbreviations for multiplicities: s = singlet, d = doublet, t = triplet, q = quartet, and m = multiplet. Data for ^13^C-NMR and ^19^F-NMR were reported in terms of chemical shifts (δ ppm). High-resolution mass spectra (HRMS) were obtained by using a Bruker Compact TOF mass spectrometer in electrospray ionization mode (ESI). Enantiomeric ratio (er) was determined by an Agilent 1260 Series HPLC utilizing DAICEL Chiralpak (AD-H, AS-H, or IC) or Chiralcel (OD-H) columns (4.6 mm × 250 mmL). Optical rotations were measured with a Perkin Elmer 343 polarimeter and were reported as: [α]_D_^T^ (concentration in g/100 mL, solvent). The NMR spectra of all new compounds and HPLC spectra of oxidative coupling products were provided in the Supplementary Materials.

### 3.2. Preparation of Ligands

**Scheme 2:**
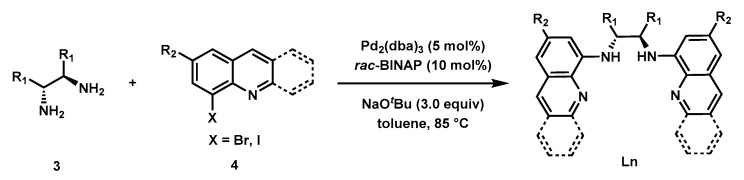
Synthesis of Bisquinolyldiamine Ligands.

General Procedure (Scheme 2): To an oven-dried Schlenk flask were added diamine **3** (1.0 equiv), Pd_2_(dba)_3_ (5 mol%), *rac*-BINAP (10 mol%), NaO*^t^*Bu (3.0 equiv), and toluene under Ar atmosphere. Then 8-haloqunoline **4** (2.2 equiv) was added directly. The flask was sealed, and the reaction was stirred at 85 °C until the complete consumption of the starting material **3**. The mixture was cooled to room temperature, filtered through a silica plug, and the plug was washed with EA. The combined filtrates were concentrated under reduced pressure, and the residue was purified by silica gel chromatography to give the desired product **Ln**.

*(1R,2R)-N^1^,N^2^-Di(quinolin-8-yl)cyclohexane-1,2-diamine* (**L1**) [53]: Following the general procedure, the reaction was carried out with (1*R*,2*R*)-cyclohexane-1,2-diamine **3a** (0.36 g, 3.2 mmol, 1.0 equiv); Pd_2_(dba)_3_ (0.15 g, 0.16 mmol, 5 mol%); *rac*-BINAP (0.20 g, 0.32 mmol, 10 mol%); NaO*^t^*Bu (0.92 g, 9.6 mmol, 3.0 equiv); and 8-bromoqunoline **4a** (1.46 g, 7.0 mmol, 2.2 equiv) in 30 mL of toluene. The desired product was obtained (0.88 g, 75% yield) as a pale yellow solid after purification by silica gel chromatography (PE/EA = 30/1 to 10/1). ^1^H-NMR (400 MHz, Chloroform-*d*) δ 8.57 (dd, *J* = 4.2, 1.7 Hz, 2H), 7.98 (dd, *J* = 8.3, 1.7 Hz, 2H), 7.36 (td, *J* = 8.0, 0.9 Hz, 2H), 7.30–7.25 (m, 2H), 6.98 (dd, *J* = 8.2, 1.3 Hz, 2H), 6.84 (dd, *J* = 7.7, 1.1 Hz, 2H), 6.43 (brs, 2H), 3.86–3.67 (m, 2H), 2.49–2.31 (m, 2H), 1.86 (td, *J* = 4.6, 4.1, 2.2 Hz, 2H), 1.67–1.48 (m, 4H).

*(1R,2R)-N^1^,N^2^-Bis(6-fluoroquinolin-8-yl)cyclohexane-1,2-diamine* (**L2**): Following the general procedure, the reaction was carried out with (1*R*,2*R*)-cyclohexane-1,2-diamine **3a** (81.6 mg, 0.7 mmol, 1.0 equiv); Pd_2_(dba)_3_ (32.8 mg, 0.04 mmol, 5 mol%); *rac*-BINAP (45.1 mg, 0.07 mmol, 10 mol%); NaO*^t^*Bu (206.9 mg, 2.2 mmol, 3.0 equiv); and 8-bromo-6-fluoroquinoline **4b** (356.7 mg, 1.6 mmol, 2.2 equiv) in 2 mL of toluene. The desired product was obtained (262.3 mg, 93% yield) as a pale yellow solid after purification by silica gel chromatography (PE/EA = 5/1). ^1^H-NMR (400 MHz, Chloroform-*d*) δ 8.49 (dd, *J =* 4.2, 1.5 Hz, 2H), 7.87 (dd, *J =* 8.3, 1.2 Hz, 2H), 7.34–7.21 (m, 2H), 6.68–6.49 (m, 4H), 6.46 (dd, *J* = 9.3, 2.5 Hz, 2H), 3.76–3.56 (m, 2H), 2.45–2.28 (m, 2H), 1.97–1.79 (m, 2H), 1.66–1.47 (m, 4H) (Figure S2); ^13^C-NMR (100 MHz, Chloroform-*d*) δ 162.4 (d, *J_C–__F_* = 243.3 Hz), 146.3 (d, *J_C–__F_* = 13.5 Hz), 145.6 (d, *J_C–__F_* = 2.4 Hz), 135.6, 135.4 (d, *J_C–__F_* = 5.8 Hz), 129.3 (d, *J_C–__F_* = 12.8 Hz), 122.2, 96.1 (d, *J_C–__F_* = 22.8 Hz), 95.1 (d, *J_C–__F_* = 30.6 Hz), 56.6, 31.7, 24.5 (Figure S3); ^19^F NMR (376 MHz, Chloroform-*d*) δ –110.9 (Figure S4); HRMS (ESI^+^) calcd for C_24_H_23_F_2_N_4_ [M + H]^+^: 405.1885, found 405.1880; ${\left[\alpha \right]}_{D}^{24}$ = −315.6 (c = 0.2, CHCl3); M. p. 162–166 °C.

*(1R,2R)-N^1^,N^2^-Bis(6-(trifluoromethyl)quinolin-8-yl)cyclohexane-1,2-diamine* (**L3**): Following the general procedure, the reaction was carried out with (1*R*,2*R*)-cyclohexane-1,2-diamine **3a** (0.31 g, 2.7 mmol, 1.0 equiv); Pd_2_(dba)_3_ (0.13 g, 0.14 mmol, 5 mol%); *rac*-BINAP (0.17 g, 0.28 mmol, 10 mol%); NaO*^t^*Bu (0.79 g, 8.2 mmol, 3.0 equiv); and 8-bromo-6-trifluoromethylquinoline **4c** (1.57 g, 5.7 mmol, 2.1 equiv) in 25 mL of toluene. The desired product was obtained (1.04 g, 76% yield) as a yellow green solid after purification by silica gel chromatography (PE/DCM = 10/1 to 5/1 to 1/1). ^1^H-NMR (400 MHz, Chloroform-*d*) δ 8.61 (dd, *J* = 4.2, 1.5 Hz, 2H), 7.95 (d, *J* = 8.1 Hz, 2H), 7.32 (dd, *J* = 8.2, 4.2 Hz, 2H), 7.14–7.03 (m, 2H), 6.95–6.83 (m, 2H), 6.55 (s, 2H), 3.87–3.62 (m, 2H), 2.45–2.24 (m, 2H), 2.03–1.85 (m, 2H), 1.70–1.48 (m, 4H) (Figure S5); ^13^C-NMR (100 MHz, Chloroform-*d*) δ 148.4, 144.9, 138.8, 136.9, 129.4 (q, *J_C–__F_* = 31.7 Hz), 127.5, 124.5 (q, *J_C–__F_* = 272.7 Hz), 122.3, 110.6 (q, *J_C–__F_* = 4.7 Hz), 100.0, 57.3, 32.3, 24.8 (Figure S6); ^19^F NMR (376 MHz, Chloroform-*d*) δ –62.8 (Figure S7); HRMS (ESI^+^) calcd for C_26_H_23_F_6_N_4_ [M + H]^+^: 505.1821, found 505.1817; ${\left[\alpha \right]}_{D}^{24}$ = −329.1 (*c* = 1.0, CHCl_3_); M. p. 120–124 °C.

*(1R,2R)-N^1^,N^2^-Bis(6-(tert-butyl)quinolin-8-yl)cyclohexane-1,2-diamine* (**L4**): Following the general procedure, the reaction was carried out with (1*R*,2*R*)-cyclohexane-1,2-diamine **3a** (0.68 g, 6.0 mmol, 1.0 equiv); Pd_2_(dba)_3_ (0.28 g, 0.3 mmol, 5 mol%); *rac*-BINAP (0.37 g, 0.6 mmol, 10 mol%); NaO*^t^*Bu (1.73 g, 18 mmol, 3.0 equiv); and 8-bromo-6-(*tert*-butyl)quinoline **4d** (3.46 g, 13.1 mmol, 2.2 equiv) in 35 mL of toluene. The desired product was obtained (2.44 g, 85% yield) as a yellow solid after purification by silica gel chromatography (PE/DCM = 10/1 to PE/EA = 5/1). ^1^H-NMR (400 MHz, Chloroform-*d*) δ 8.55 (dd, *J* = 4.3, 1.7 Hz, 2H), 7.99 (d, *J* = 7.5 Hz, 2H), 7.31–7.25 (m, 2H), 7.02–6.89 (m, 4H), 3.87–3.81 (m, 2H), 2.38 (d, *J* = 12.2 Hz, 2H), 1.91–1.83 (m, 2H), 1.71–1.50 (m, 4H), 1.36 (s, 18H) (Figure S8); ^13^C-NMR (100 MHz, Chloroform-*d*) δ 150.6, 146.2, 143.5, 137.4, 136.0, 128.5, 121.3, 109.4, 104.3, 55.5, 35.2, 31.4, 30.7, 24.0 (Figure S9); HRMS (ESI^+^) calcd for C_32_H_41_N_4_ [M + H]^+^: 481.3326, found 481.3323; ${\left[\alpha \right]}_{D}^{24}$ = −39.2 (*c* = 1.0, CHCl_3_); M. p. 172–174 °C.

*(1R,2R)-N^1^,N^2^-Di(acridin-4-yl)cyclohexane-1,2-diamine* (**L5**): Following the general procedure, the reaction was carried out with (1*R*,2*R*)-cyclohexane-1,2-diamine **3a** (0.19 g, 1.6 mmol, 1.0 equiv); Pd_2_(dba)_3_ (0.08 g, 0.08 mmol, 5 mol%); *rac*-BINAP (0.10 g, 0.16 mmol, 10 mol%); NaO*^t^*Bu (0.47 g, 4.9 mmol, 3.0 equiv); and 4-iodoacridine **4e** (1.07 g, 3.5 mmol, 2.2 equiv) in 30 mL of toluene. The desired product was obtained (0.46 g, 61% yield) as a yellow solid after purification by silica gel chromatography (PE/DCM = 2/1 to PE/DCM = 1/1 to DCM). ^1^H-NMR (400 MHz, DMSO-*d*_6_) δ 8.78 (s, 2H), 8.01 (d, *J* = 8.3 Hz, 2H), 7.91 (d, *J* = 8.7 Hz, 2H), 7.67 (t, *J* = 8.0 Hz, 2H), 7.49 (t, *J* = 8.0 Hz, 2H), 7.43 (t, *J* = 7.9 Hz, 2H), 7.18 (d, *J* = 8.4 Hz, 2H), 6.97 (d, *J* = 7.5 Hz, 2H), 6.75 (d, *J* = 7.0 Hz, 2H), 3.98–3.90 (m, 2H), 2.37–2.30 (m, 2H), 1.86–1.80 (m, 2H), 1.62–1.55 (m, 4H) (Figure S10); ^13^C-NMR (100 MHz, Chloroform-*d*) δ 146.5, 144.2, 140.7, 135.1, 129.7, 128.9, 127.8, 127.3, 127.2, 127.0, 125.5, 113.8, 103.1, 56.6, 31.6, 24.4 (Figure S11); HRMS (ESI^+^) calcd for C_32_H_29_N_4_ [M + H]^+^: 469.2387, found 469.2371; ${\left[\alpha \right]}_{D}^{24}$ = −678.0 (*c* = 0.5, CHCl_3_); M. p. 198–202 °C.

*(R)-N^2^,N^2′^-Di(quinolin-8-yl)-[1,1′-binaphthalene]-2,2′-diamine* (**L6**) [53]: Following the general procedure, the reaction was carried out with (*R*)-[1,1′-binaphthalene]-2,2′-diamine **3b** (141.9 mg, 0.5 mmol, 1.0 equiv); Pd_2_(dba)_3_ (23.2 mg, 0.025 mmol, 5 mol%); *rac*-BINAP (31.4 mg, 0.05 mmol, 10 mol%); NaO*^t^*Bu (148.2 mg, 1.5 mmol, 3.0 equiv); and 8-bromoqunoline **4a** (224.0 mg, 1.1 mmol, 2.2 equiv) in 10 mL of toluene. The desired product was obtained (196.6 mg, 73% yield) as a yellow solid after purification by recrystallization from EA. ^1^H-NMR (400 MHz, Chloroform-*d*) δ 8.42 (d, *J* = 3.0 Hz, 2H), 7.99–7.95 (m, 4H), 7.94–7.86 (m, 6H), 7.37 (dt, *J* = 8.0, 4.0 Hz, 2H), 7.30–7.19 (m, 8H), 6.94–6.89 (m, 4H).

*(12R)-N^11^,N^12^-Di(quinolin-8-yl)-9,10-dihydro-9,10-ethanoanthracene-11,12-diamine* (**L7**) [53]: Following the general procedure, the reaction carried out with (12*R*)-9,10-dihydro-9,10-ethanoanthracene-11,12-diamine **3c** (20 mg, 0.08 mmol, 1.0 equiv); Pd_2_(dba)_3_ (5.3 mg, 5 mol%); *rac*-BINAP (6.2 mg, 10 mol%); NaO*^t^*Bu (25.5 mg, 0.26 mmol, 3.0 equiv); and 8-bromoqunoline **4a** (41.8 mg, 0.2 mmol, 2.2 equiv) in 1 mL of toluene. The desired product was obtained (33.4 mg, 85% yield) as a white solid after purification by silica gel chromatography (PE/EA = 5/1). ^1^H-NMR (400 MHz, Chloroform-*d*) δ 8.61 (dd, *J* = 4.1, 1.4 Hz, 2H), 8.06 (d, *J* = 4.0 Hz, 2H), 7.44 (d, *J* = 7.1 Hz, 2H), 7.40–7.13 (m, 10H), 7.06 (d, *J* = 8.1 Hz, 2H), 6.91 (d, *J* = 8.0 Hz, 2H), 6.18 (s, 2H), 4.63 (s, 2H), 3.97 (s, 2H).

*(1R,2R)-1,2-Diphenyl-N^1^,N^2^-di(quinolin-8-yl)ethane-1,2-diamine* (**L8**) [58]: Following the general procedure, the reaction was carried out with (1*R*,2*R*)-1,2-diphenylethane-1,2-diamine **3d** (1.06 g, 5.0 mmol, 1.0 equiv); Pd_2_(dba)_3_ (0.23 g, 0.25 mmol, 5 mol%); *rac*-BINAP (0.33 g, 0.5 mmol, 10 mol%); NaO*^t^*Bu (1.47 g, 15 mmol, 3.0 equiv); and 8-bromoqunoline **4a** (2.51 g, 12 mmol, 2.4 equiv) in 90 mL of toluene. The desired product was obtained (1.47 g, 63% yield) as a white solid after purification by silica gel chromatography (PE/DCM = 30/1 to PE/EA = 5/1). ^1^H-NMR (400 MHz, Chloroform-*d*) δ 8.67 (dd, *J* = 4.3, 1.6 Hz, 2H), 8.05 (d, *J* = 8.4 Hz, 2H), 7.36 (m, 4H), 7.28–7.09 (m, 12H), 7.03 (d, *J* = 8.0 Hz, 2H), 6.50 (d, *J* = 7.6 Hz, 2H), 5.01 (s, 2H).

### 3.3. Preparation of Substituted Quinolines

**Scheme 3:**
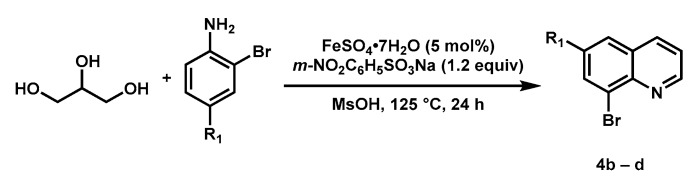
Synthesis of Substituted Quinolines (Skraup Reaction).

General procedure for synthesis of substituted 8-bromoquinoline (Scheme 3): to a 50 mL round bottom flask was added 4-substituted 2-bromoaniline**,** glycerol (17.0 equiv), *m*-nitrobenzenesulfonate sodium (1.2 equiv), FeSO_4_•7H_2_O (0.05 equiv), and MsOH. The reaction mixture was heated at 125 °C for 24 h. After cooling to room temperature, aqueous NaOH solution (2.5 M) was added to the reaction mixture to adjust pH to 12. Then EtOH was added to form a black solution, which was extracted with EA or DCM (3 × 100 mL). The combined organic phase was washed with H_2_O (100 mL), brine (100 mL), and dried with anhydrous Na_2_SO_4_. After removing the solvents, the residue was purified by silica gel chromatography.

*8-Bromo-6-fluoroquinoline* (**4b**) [59]: Following the general procedure, the reaction was carried out with 2-bromo-4-fluoroaniline (1.57 g, 8.3 mmol, 1.0 equiv); glycerol (11 mL, 149.0 mmol, 18.0 equiv); *m*-nitrobenzenesulfonate sodium (2.24 g, 10.0 mmol, 1.2 equiv); FeSO_4_•7H_2_O (0.12 g, 0.4 mmol, 0.05 equiv), and MsOH (11 mL). The desired product was obtained (0.86 g, 43% yield) as a pale yellow solid after purification by silica gel chromatography (PE/EA = 10/1 to 5/1). ^1^H-NMR (400 MHz, Chloroform-*d*) δ 9.02 (dd, *J* = 4.2, 1.2 Hz, 1H), 8.14 (dd, *J* = 8.3, 1.6 Hz, 1H), 7.90 (dd, *J* = 8.1, 2.7 Hz, 1H), 7.50 (ddd, *J* = 8.3, 4.2, 0.6 Hz, 1H), 7.46 (dd, *J* = 8.3, 2.7 Hz, 1H).

*8-Bromo-6-(trifluoromethyl)quinoline* (**4c**): Following the general procedure, the reaction was carried out with 2-bromo-4-(trifluoromethyl)aniline (6.38 g, 26.6 mmol, 1.0 equiv); glycerol (20 mL, 271.5 mmol, 10.0 equiv); *m*-nitrobenzenesulfonate sodium (7.19 g, 32.0 mmol, 1.2 equiv); FeSO_4_•7H_2_O (0.37 g, 1.3 mmol, 0.05 equiv), and MsOH (35 mL). The desired product was obtained (1.57 g, 21% yield) as a pale orange solid after purification by silica gel chromatography (PE/EA = 10/1 to 5/1). ^1^H-NMR (400 MHz, Chloroform-*d*) δ 9.16 (dd, *J* = 4.2, 1.7 Hz, 1H), 8.28 (dd, *J* = 8.3, 1.7 Hz, 1H), 8.24 (d, *J* = 1.9 Hz, 1H), 8.16–8.10 (m, 1H), 7.60 (dd, *J* = 8.3, 4.2 Hz, 1H) (Figure S12); ^13^C-NMR (100 MHz, Chloroform-*d*) δ 153.4, 146.5, 137.7, 129.1(q, *J_C–__F_* = 33.4 Hz), 129.0(q, *J_C–__F_* = 3.1 Hz), 128.4, 126.3, 125.7 (q, *J_C–__F_* = 4.3 Hz), 123.2, 123.2(q, *J_C–__F_* = 272.8 Hz) (Figure S13); ^19^F NMR (376 MHz, Chloroform-*d*) δ –62.5 (Figure S14); HRMS (ESI^+^) calcd for C_10_H_6_BrF_3_N [M + H]^+^: 275.9630, found 275.9620; M. p. 58–62 °C.

*8-Bromo-6-(tert-butyl)quinoline* (**4d**) [60]: Following the general procedure, the reaction was carried out with 2-bromo-4-(*tert*-butyl)aniline (1.78 g, 7.8 mmol, 1.0 equiv); glycerol (10 mL, 135.7 mmol, 17.0 equiv); *m*-nitrobenzenesulfonate sodium (2.11 g, 9.4 mmol, 1.2 equiv); FeSO_4_•7H_2_O (0.11 g, 0.41 mmol, 0.05 equiv); and MsOH (10 mL). The desired product was obtained (1.73 g, 84% yield) as a yellow solid after purification by silica gel chromatography (PE/EA = 20/1). ^1^H-NMR (400 MHz, Chloroform-*d*) δ 9.00 (dd, *J* = 4.2, 1.5 Hz, 1H), 8.18–8.12 (m, 2H), 7.70 (d, *J* = 2.0 Hz, 1H), 7.45 (dd, *J* = 8.2, 4.2 Hz, 1H), 1.42 (s, 9H).

**Scheme 4:**
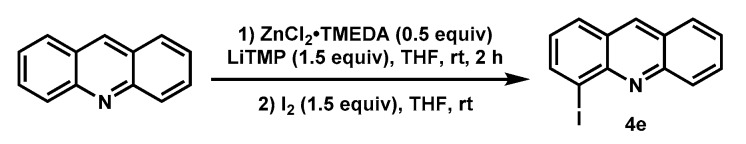
Synthesis of 4-Iodoacridine.

4-Iodoacridine [**4e**] (Scheme 4)

To a 100 mL Schlenk flask were added TMP (1.14 g, 8.1 mmol, 1.5 equiv) and 20 mL of THF. The solution was cooled to 0 °C, and *^n^*BuLi (2.4 M, 4 mL, 9.6 mmol, 1.7 equiv) was added dropwise by syringe. Upon the completion of the addition, the mixture was stirred at 0 °C for another 30 min. Then ZnCl_2_•TMEDA (0.68 g, 2.7 mmol, 0.5 equiv) was added at 0 °C, and the resultant mixture was stirred for 20 min before acridine (0.98 g, 5.5 mmol, 1.0 equiv) was added. After the reaction was warmed up to 25 °C, I_2_ (2.17 g, 8.5 mmol, 1.5 equiv) in THF (20 mL) was added dropwise. The reaction mixture was stirred for 2 h and then quenched with saturated Na_2_S_2_O_3_ solution and extracted with EA (3 × 30 mL). The combined organic phase was washed with brine and dried over anhydrous Na_2_SO_4_. The desired product was obtained (1.08 g, 64% yield) as a yellow solid after purification by silica gel chromatography (PE/DCM = 20/1 to 5/1). ^1^H-NMR (400 MHz, Chloroform-*d*) δ 8.72 (s, 1H), 8.46 (dd, *J* = 7.1, 1.1 Hz, 1H), 8.39 (d, *J* = 8.8 Hz, 1H), 8.09–7.93 (m, 2H), 7.83 (ddd, *J* = 8.5, 6.6, 1.3 Hz, 1H), 7.63–7.52 (m, 1H), 7.31–7.19 (m, 1H) (Figure S15); ^13^C-NMR (100 MHz, Chloroform-*d*) δ 149.8, 147.1, 141.0, 137.2, 130.9, 130.1, 129.4, 127.9, 127.2, 126.7, 126.63, 126.58, 104.0 (Figure S16); HRMS (ESI^+^) calcd for C_13_H_9_IN [M + H]^+^: 305.9774, found 305.9763; M.p. 100–104 °C.

*2-Methoxynaphthalene* [61]: Following the reported procedure, the reaction was carried out with 2-naphthol (**1.14** g, 10 mmol, 1.0 equiv); NaH (60% wt, 0.41 g, 17 mmol, 1.7 equiv); and MeI (1.76 g, 12 mmol, 1.2 equiv) in 10 mL of DMF. The desired product was obtained (1.28 g, 81% yield) as a white solid after purification by silica gel chromatography (DCM). ^1^H-NMR (400 MHz, Chloroform-*d*) δ 7.78 (dd, *J* = 11.5, 8.4 Hz, 3H), 7.47 (ddd, *J* = 8.1, 6.8, 1.3 Hz, 1H), 7.36 (ddd, *J* = 8.1, 6.8, 1.2 Hz, 1H), 7.22–7.13 (m, 2H), 3.94 (s, 3H).

*2-Bromo-3-methoxynaphthalene* [62]: Following the reported procedure, the reaction was carried out with 2-methoxynaphthalene (0.79 g, 5.0 mmol, 1.0 equiv); *^n^*BuLi solution (1.67 M in hexane, 3 mL, 5.3 mmol, 1.1 equiv); and 1,2-dibromoethane (1.30 g, 6.9 mmol, 1.3 equiv) in 10 mL of THF. The desired product was obtained (0.87 g, 73% yield) as a white solid after recrystallization from hot hexane for 3 times. ^1^H-NMR (400 MHz, Chloroform-*d*) δ 8.06 (s, 1H), 7.71 (dd, *J* = 13.0, 8.2 Hz, 2H), 7.51–7.42 (m, 1H), 7.40–7.32 (m, 1H), 7.16 (s, 1H), 4.01 (s, 3H).

*3-Methoxynaphthalen-2-ol* (1b) [63]: Following the reported procedure, the reaction was carried out with naphthalene-2, 3-diol (1.60 g, 10 mmol, 1.0 equiv); K_2_CO_3_ (1.81 g, 13 mmol, 1.3 equiv); and MeI (1.73 g, 12 mmol, 1.2 equiv) in 10 mL of acetone. The desired product was obtained (0.57 g, 33% yield) as a white solid after purification by silica gel chromatography (PE/EA = 50/1 to PE/EA = 20/1 to PE/EA = 10/1). ^1^H-NMR (400 MHz, Chloroform-*d*) δ 8.06 (s, 1H), 7.71 (dd, *J* = 13.0, 8.2 Hz, 2H), 7.50–7.42 (m, 1H), 7.40–7.33 (m, 1H), 7.16 (s, 1H), 4.01 (s, 3H).

*3-(Benzyloxy)naphthalen-2-ol* (1c) [64]: Following the reported procedure, the reaction was carried out with naphthalene-2, 3-diol (1.61 g, 10 mmol, 1.0 equiv); K_2_CO_3_ (1.82 g, 13 mmol, 1.3 equiv); and BnBr (2.58 g, 15 mmol, 1.5 equiv) in 20 mL of DMF. The desired product was obtained (0.88 g, 35% yield) as a yellow solid after purification by silica gel chromatography (PE/EA = 50/1 to 20/1 to 10/1). ^1^H-NMR (400 MHz, Chloroform-*d*) δ 7.71–7.64 (m, 2H), 7.52–7.31 (m, 7H), 7.29 (s, 1H), 7.22 (s, 1H), 5.97 (s, 1H), 5.24 (s, 2H).

**Scheme 5:**
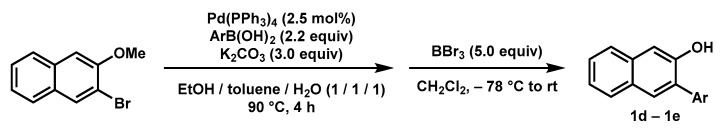
Synthesis of Substrates **1d**–**1e**.

General procedure (Scheme 5): To a Schlenk flask were added 2-bromo-3-methoxynaphthalene (1.0 equiv), aryl boronic acid (2.2 equiv), K_2_CO_3_ (3.0 equiv), Pd(PPh_3_)_4_ (2.5 mol%), and degassed EtOH/toluene/water (1/1/1) under Ar atmosphere. The mixture was heated at 90 °C until the completion of the reaction. Then the mixture was cooled to room temperature, and DCM was added. The mixture was washed with NaOH solution (20% wt), and the aqueous phase was extracted with DCM (2 × 20 mL). The combined organic phase was washed with brine (20 mL) and dried over anhydrous MgSO_4_. After removing the solvent, the residue was dissolved in anhydrous DCM. The solution was cooled to −78 °C, and BBr_3_ (1 M in DCM, 5.0 equiv) was added slowly by syringe. Then the mixture was warmed up to room temperature and stirred until the complete consumption of the starting material. The mixture was poured into the ice water (50 mL) and extracted with DCM (3 × 50 mL). The combined organic phase was washed with brine (100 mL) and dried over anhydrous Na_2_SO_4_. After removing the solvent, the residue was purified by silica gel chromatography to give the desired product.

*3-(o-Tolyl)naphthalen-2-ol* (**1d**) [65]: Following the general procedure, the reaction was carried out with 2-bromo-3-methoxynaphthalene (236.2 mg, 1.0 mmol, 1.0 equiv); *o*-tolylboronic acid (304.8 mg, 2.2 mmol, 2.2 equiv); K_2_CO_3_ (417.7 mg, 3.0 mmol, 3.0 equiv); and Pd(PPh_3_)_4_ (29.9 mg, 2.5 mol%) in 6 mL of degassed solvents. Then BBr_3_ (1 M in DCM, 5 mL, 5 mmol, 5.0 equiv) was used to remove the methyl group. The desired product was obtained (185.1 mg, 79% yield overall) as a brown sticky liquid after purification by silica gel chromatography (PE/DCM = 10/1). ^1^H-NMR (400 MHz, Chloroform-*d*) δ 7.80–7.72 (m, 2H), 7.63 (s, 1H), 7.45 (ddd, *J* = 8.3, 6.9, 1.2 Hz, 1H), 7.44–7.28 (m, 6H), 4.92 (s, 1H), 2.20 (s, 3H).

*3-Phenylnaphthalen-2-ol* (**1e**) [66]: Following the general procedure, the reaction was carried out with 2-bromo-3-methoxynaphthalene (0.71 g, 3.0 mmol, 1.0 equiv); phenylboronic acid (0.55 g, 4.5 mmol, 1.5 equiv); K_2_CO_3_ (1.90 g, 13.8 mmol, 4.5 equiv); and Pd(PPh_3_)_4_ (0.09 g, 2.5 mol%) in 30 mL of degassed solvent. Then BBr_3_ (1 M in DCM, 15 mL, 15 mmol, 5.0 equiv) was used to remove the methyl group. The desired product was obtained (0.63 g, 95% yield overall) as a pale brown solid after purification by silica gel chromatography (DCM). ^1^H-NMR (400 MHz, Chloroform-*d*) δ 7.81–7.76 (m, 1H), 7.74 (d, *J* = 7.2 Hz, 2H), 7.60–7.51 (m, 4H), 7.49–7.41 (m, 2H), 7.39–7.32 (m, 2H), 5.30 (s, 1H).

*3-Bromonaphthalen-2-ol* (**1f**) [67]: Following the general procedure, the reaction was carried out with 2-bromo-3-methoxynaphthalene (240.6 mg, 1.0 mmol, 1.0 equiv) and BBr_3_ (1 M in DCM, 5 mL, 5.0 mmol, 5.0 equiv). The desired product was obtained (226.0 mg, quantitative yield) as a white solid after purification by silica gel chromatography (DCM). ^1^H-NMR (400 MHz, Chloroform-*d*) δ 8.03 (s, 1H), 7.69 (ddt, *J* = 7.4, 2.2, 1.2 Hz, 2H), 7.45 (ddd, *J* = 8.2, 6.8, 1.3 Hz, 1H), 7.39 (s, 1H), 7.35 (ddd, *J* = 8.2, 6.8, 1.2 Hz, 1H), 5.64 (s, 1H).

*6-Phenylnaphthalen-2-ol* (**1k**) [5]: Following the general procedure, the reaction was carried out with 6-bromonaphthalen-2-ol (1.12 g, 5.0 mmol, 1.0 equiv); phenylboronic acid (0.73 g, 6.0 mmol, 1.2 equiv); K_2_CO_3_ (3.00 g, 21.8 mmol, 4.4 equiv); and Pd(PPh_3_)_4_ (0.15 g, 2.5 mol%) in 30 mL of degassed solvent. The desired product was obtained (0.77 g, 70% yield) as a white solid after purification by silica gel chromatography (DCM). ^1^H-NMR (400 MHz, Chloroform-*d*) δ 7.98 (d, *J* = 1.7 Hz, 1H), 7.82 (d, *J* = 8.8 Hz, 1H), 7.76 (d, *J* = 8.5 Hz, 1H), 7.71 (ddd, *J* = 8.2, 2.8, 1.5 Hz, 3H), 7.48 (dd, *J* = 8.5, 6.9 Hz, 2H), 7.41–7.33 (m, 1H), 7.18 (d, *J* = 2.5 Hz, 1H), 7.14 (dd, *J* = 8.8, 2.5 Hz, 1H).

*7-(Benzyloxy)naphthalen-2-ol* (**1l**) [68]: Following the reported procedure, the reaction was carried out with naphthalene-2,7-diol (1.60 g, 10 mmol, 1.0 equiv); K_2_CO_3_ (1.80 g, 13 mmol, 1.3 equiv), and BnBr (2.65 g, 15 mmol, 1.5 equiv) in 20 mL of DMF. The desired product was obtained (0.75 g, 30% yield) as a white solid after purification by silica gel chromatography (PE/EA = 5/1). ^1^H-NMR (400 MHz, Chloroform-*d*) δ 7.71–7.63 (m, 2H), 7.52–7.45 (m, 2H), 7.45–7.38 (m, 2H), 7.38–7.31 (m, 1H), 7.15–7.06 (m, 2H), 7.04 (d, *J* = 2.5 Hz, 1H), 6.94 (dd, *J* = 8.8, 2.4 Hz, 1H), 5.16 (s, 2H).

*7-Butoxynaphthalen-2-ol* (**1m**) [69]: Following the reported procedure, the reaction was carried out with naphthalene-2,7-diol (1.60 g, 10 mmol, 1.0 equiv); K_2_CO_3_ (1.80 g, 13 mmol, 1.3 equiv); and *^n^*BuI (2.26 g, 12 mmol, 1.2 equiv) in 20 mL of acetone. The desired product was obtained (0.32 g, 15% yield) as a white solid after purification by silica gel chromatography (PE/DCM = 1/1 to PE/EA = 5/1). ^1^H-NMR (400 MHz, Chloroform-*d*) δ 7.65 (dd, *J* = 8.7, 2.5 Hz, 2H), 7.17–6.83 (m, 4H), 5.04 (s, 1H), 4.06 (t, *J* = 6.5 Hz, 2H), 1.83 (dq, *J* = 8.7, 6.6 Hz, 2H), 1.62–1.43 (m, 2H), 1.00 (t, *J* = 7.4 Hz, 3H);

*7-((tert-Butyldimethylsilyl)oxy)naphthalen-2-ol* (**1n**) [70]**:** Following the reported procedure, the reaction was carried out with naphthalene-2,7-diol (1.60 g, 10 mmol, 1.0 equiv); imidazole (0.68 g, 10 mmol, 1.0 equiv); and TBSCl (1.35 g, 9 mmol, 0.9 equiv) in 15 mL of DMF. The desired product was obtained (0.75 g, 33% yield) as a yellow solid after purification by silica gel chromatography (PE/EA = 5/1). ^1^H-NMR (400 MHz, Chloroform-*d*) δ 7.65 (t, *J* = 9.3 Hz, 1H), 7.03 (d, *J* = 2.2 Hz, 1H), 7.00 (d, *J* = 2.4 Hz, 1H), 6.93 (ddd, *J* = 11.0, 8.8, 2.4 Hz, 1H), 1.01 (s, 4H), 0.24 (s, 3H).

*7-Methoxynaphthalen-2-ol* (**1o**) [69]: Following the reported procedure, the reaction was carried out with naphthalene-2,7-diol (1.61 g, 10 mmol, 1.0 equiv); K_2_CO_3_ (1.80 g, 13 mmol, 1.3 equiv); and MeI (1.75 g, 12 mmol, 1.2 equiv) in 20 mL of acetone. The desired product was obtained (0.53 g, 30% yield) as a white solid after purification by silica gel chromatography (PE/DCM = 1/1 to PE/EA = 5/1). ^1^H-NMR (400 MHz, Chloroform-*d*) δ 7.66 (dd, *J* = 9.2, 3.6 Hz, 2H), 7.06 (d, *J* = 2.3 Hz, 1H), 7.01–6.97 (m, 2H), 6.94 (dd, *J* = 8.7, 2.4 Hz, 1H), 3.90 (s, 3H).

### 3.4. Iron-Catalyzed Asymmetric Oxidative Coupling Reaction of 2-Naphthols

*(S)-[1,1′-Binaphthalene]-2,2′-diol* (**2a**) [47]: Fe(ClO_4_)_2_ (12.7 mg, 10 mol%; NOTE: perchlorate salt is a potential explosive [71] and should be handled with extreme caution) and **L1** (9.2 mg, 5 mol%) were dissolved in anhydrous PhCl (5 mL) in a 25 mL Schlenk tube, and the mixture was stirred at room temperature for 30 min. Then, 2-naphthol (72.3 mg, 0.5 mmol, 1.0 equiv) and MS 4Å (152.7 mg) were added. The reaction mixture was quickly evacuated and refilled with oxygen (1 atm), and this operation was repeated for three cycles. Then the mixture was stirred at 50 °C under oxygen, as monitored by TLC. The desired product was obtained (60.6 mg, 84% yield) as a pale yellow solid after purification by silica gel chromatography (PE to PE/EA = 10/1 to 5/1). 80:20 er (HPLC: Chiralpak AS-H, hexane/propan-2-ol = 90/10, 0.5 mL/min, λ = 230 nm, t*_R_* (min): major = 24.9, minor = 38.9). ^1^H-NMR (400 MHz, Chloroform-*d*) δ 7.99 (d, *J* = 8.9 Hz, 2H), 7.90 (d, *J* = 7.9 Hz, 2H), 7.38 (td, *J* = 7.7, 1.6 Hz, 4H), 7.31 (ddd, *J* = 8.2, 6.9, 1.4 Hz, 2H), 7.19–7.12 (m, 2H), 5.04 (s, 2H).

*(S)-3,3′-Dimethoxy-[1,1′-binaphthalene]-2,2′-diol* (**2b**) [30]: The reaction was conducted with Fe(ClO_4_)_2_ (12.7 mg, 10 mol%) and **L1** (9.2 mg, 5 mol%); 3-methoxynaphthalen-2-ol (87.5 mg, 0.5 mmol, 1.0 equiv); and MS 4Å (152.5 mg). The desired product was obtained (76.2 mg, 88% yield) as a white solid after purification by silica gel chromatography (PE to PE/EA = 5/1). 81:19 er (HPLC: Chiralpak AS-H, hexane/propan-2-ol = 50/50, 1.0 mL/min, λ = 230 nm, t*_R_* (min): major = 14.3, minor = 24.4). ^1^H-NMR (400 MHz, Chloroform-*d*) δ 7.83–7.74 (m, 2H), 7.38–7.28 (m, 4H), 7.23–7.08 (m, 4H), 5.90 (s, 2H), 4.10 (s, 6H).

*(S)-3,3′-Bis(benzyloxy)-[1,1′-binaphthalene]-2,2′-diol* (**2c**) [72]: The reaction was conducted with Fe(ClO_4_)_2_ (12.7 mg, 10 mol%) and **L1** (9.2 mg, 5 mol%); 3-(benzyloxy)naphthalen-2-ol (125.2 mg, 0.5 mmol, 1.0 equiv); and MS 4Å (150.0 mg). The desired product was obtained (84.9 mg, 68% yield) as a white solid after purification by silica gel chromatography (PE to PE/EA = 5/1). 80:20 er (HPLC: Chiralpak AS-H, hexane/propan-2-ol = 85/15, 1.0 mL/min, λ = 254 nm, t*_R_* (min): major = 35.1, minor = 42.1). ^1^H-NMR (400 MHz, Chloroform-*d*) δ 7.77 (d, *J* = 8.1 Hz, 2H), 7.57–7.48 (m, 4H), 7.47–7.36 (m, 8H), 7.32 (dt, *J* = 8.1, 4.0 Hz, 2H), 7.17 (d, *J* = 3.9 Hz, 4H), 6.01 (s, 2H), 5.33 (s, 4H).

*(S)-3,3′-Di-o-tolyl-[1,1′-binaphthalene]-2,2′-diol* (**2d**) [73]: The reaction was conducted with Fe(ClO_4_)_2_ (13.4 mg, 10 mol%) and **L1** (9.4 mg, 5 mol%); 3-(*o*-tolyl)naphthalen-2-ol (116.0 mg, 0.5 mmol, 1.0 equiv); and MS 4Å (155.6 mg). The desired product was obtained (84.2 mg, 72% yield) as a brown solid after purification by silica gel chromatography (PE/DCM = 10/1 to 1/1 to PE/EA = 5/1). 77:23 er (HPLC: Chiralpak AD-H, hexane/propan-2-ol = 70/30, 0.8 mL/min, λ = 254 nm, t*_R_* (min): major = 19.8, minor = 6.4). ^1^H-NMR (400 MHz, Chloroform-*d*) δ 7.94–7.86 (m, 2H), 7.86 (s, 2H), 7.46–7.25 (m, 14H), 5.15 (s, 2H), 2.27 (s, 6H).

*(S)-3,3′-Diphenyl-[1,1′-binaphthalene]-2,2′-diol* (**2e**) [74]: The reaction was conducted with Fe(ClO_4_)_2_ (13.1 mg, 10 mol%) and **L1** (9.0 mg, 5 mol%); 3-phenylnaphthalen-2-ol (111.6 mg, 0.5 mmol, 1.0 equiv); and MS 4Å (158.0 mg). The desired product was obtained (72.2 mg, 66% yield) as a pale yellow solid after purification by silica gel chromatography (PE/DCM = 1/1). 56:44 er (HPLC: Chiralpak IC, hexane/propan-2-ol = 90/10, 0.8 mL/min, λ = 230 nm, t*_R_* (min): major = 6.6, minor = 10.1). ^1^H-NMR (400 MHz, Chloroform-*d*) δ 8.03 (s, 2H), 7.97–7.89 (m, 2H), 7.78–7.70 (m, 4H), 7.53–7.47 (m, 4H), 7.44–7.36 (m, 4H), 7.36–7.30 (m, 2H), 7.23 (dd, *J* = 8.3, 1.1 Hz, 2H), 5.38 (s, 2H).

*(S)-3,3′-Dibromo-[1,1′-binaphthalene]-2,2′-diol* (**2f**) [75]: The reaction was conducted with Fe(ClO_4_)_2_ (12.7 mg, 10 mol%) and **L1** (9.2 mg, 5 mol%); 3-bromonaphthalen-2-ol (111.5 mg, 0.5 mmol, 1.0 equiv); and MS 4Å (150.4 mg). The desired product was obtained (56.2 mg, 51% yield) as a pale yellow solid after purification by silica gel chromatography (PE to PE/EA = 10/1). 66:34 er (HPLC: Chiralpak IC, hexane/propan-2-ol = 97/3, 1.0 mL/min, λ = 230 nm, t*_R_* (min): major = 12.2, minor = 14.3). ^1^H-NMR (400 MHz, Chloroform-*d*) δ 8.25 (d, *J* = 0.7 Hz, 2H), 7.86–7.77 (m, 2H), 7.39 (ddd, *J* = 8.2, 6.8, 1.3 Hz, 2H), 7.31 (ddd, *J* = 8.3, 6.8, 1.4 Hz, 2H), 7.10 (dq, *J* = 7.7, 0.7 Hz, 2H), 5.55 (s, 2H).

*(S)-6,6′-Dibromo-[1,1′-binaphthalene]-2,2′-diol* (**2j**) [76]: The reaction was conducted with Fe(ClO_4_)_2_ (12.7 mg, 10 mol%) and **L1** (9.2 mg, 5 mol%); 6-bromonaphthalen-2-ol (112.7 mg, 0.5 mmol, 1.0 equiv); and MS 4Å (156.0 mg). The desired product was obtained (90.6 mg, 82% yield) as a pale yellow solid after purification by silica gel chromatography (PE to PE/EA = 10/1 to 5/1). 79:21 er (HPLC: Chiralpak AS-H, hexane/propan-2-ol = 90/10, 0.5 mL/min, λ = 254 nm, t*_R_* (min): major = 27.8, minor = 38.9). ^1^H-NMR (400 MHz, Chloroform-*d*) δ 8.25 (d, *J* = 0.7 Hz, 2H), 7.85–7.78 (m, 2H), 7.39 (ddd, *J* = 8.2, 6.8, 1.3 Hz, 2H), 7.31 (ddd, *J* = 8.3, 6.8, 1.4 Hz, 2H), 7.10 (dq, *J* = 7.6, 0.7 Hz, 2H), 5.55 (s, 2H).

*(S)-6,6′-Diphenyl-[1,1′-binaphthalene]-2,2′-diol* (**2k**) [23]: The reaction was conducted with Fe(ClO_4_)_2_ (12.7 mg, 10 mol%) and **L1** (9.2 mg, 5 mol%); 6-phenylnaphthalen-2-ol (109.5 mg, 0.5 mmol, 1.0 equiv); and MS 4Å (160.6 mg). The desired product was obtained (70.2 mg, 64% yield) as pale yellow solid after purification by silica gel chromatography (PE to PE/EA = 10/1 to 5/1). 74:26 er (HPLC: Chiralpak AS-H, hexane/propan-2-ol = 90/10, 0.8 mL/min, λ = 254 nm, t*_R_* (min): major = 13.1, minor = 10.2). ^1^H-NMR (400 MHz, Chloroform-*d*) δ 8.03 (s, 2H), 7.96–7.90 (m, 2H), 7.77–7.71 (m, 4H), 7.53–7.46 (m, 4H), 7.45–7.36 (m, 4H), 7.35–7.29 (m, 2H), 7.23 (dd, *J* = 8.3, 1.1 Hz, 2H), 5.38 (s, 2H).

*(S)-7,7′-Bis(benzyloxy)-[1,1′-binaphthalene]-2,2′-diol* (**2l**) [77]: The reaction was conducted with Fe(ClO_4_)_2_ (12.9 mg, 10 mol%) and **L1** (9.0 mg, 5 mol%); 7-(benzyloxy)naphthalen-2-ol (125.4 mg, 0.5 mmol, 1.0 equiv); and MS 4Å (160.6 mg). The desired product was obtained (88.5 mg, 71% yield) as a pale yellow solid after purification by silica gel chromatography (PE to PE/EA = 5/1). 58:42 er (HPLC: Chiralcel OD-H, hexane/propan-2-ol = 85/15, 1.0 mL/min, λ = 230 nm, t*_R_* (min): major = 14.8, minor = 26.7). ^1^H-NMR (400 MHz, Chloroform-*d*) δ 7.89 (d, *J* = 8.8 Hz, 2H), 7.80 (d, *J* = 8.9 Hz, 2H), 7.22 (dt, *J* = 6.1, 2.2 Hz, 8H), 7.16 (dd, *J* = 6.6, 3.1 Hz, 4H), 7.11 (dd, *J* = 8.9, 2.5 Hz, 2H), 6.49 (d, *J* = 2.4 Hz, 2H), 4.99 (s, 2H), 4.80 (d, *J* = 11.7 Hz, 2H), 4.74 (d, *J* = 11.7 Hz, 2H).

*(S)-7,7′-Dibutoxy-[1,1′-binaphthalene]-2,2′-diol* (**2m**) [29]: The reaction was conducted with Fe(ClO_4_)_2_ (12.3 mg, 10 mol%) and **L1** (9.0 mg, 5 mol%); 7-butoxynaphthalen-2-ol (108.5 mg, 0.5 mmol, 1.0 equiv); and MS 4Å (150.0 mg). The desired product was obtained (106.5 mg, 99% yield) as a white solid after purification by silica gel chromatography (PE/EA = 10/1 to 5/1). 60:40 er (HPLC: Chiralpak AD-H, hexane/propan-2-ol = 90/10, 1.0 mL/min, λ = 254 nm, t*_R_* (min): major = 8.3, minor = 19.3). ^1^H-NMR (400 MHz, Chloroform-*d*) δ 7.84 (d, *J* = 8.8 Hz, 2H), 7.76 (d, *J* = 8.9 Hz, 2H), 7.20 (d, *J* = 8.8 Hz, 2H), 7.03 (dd, *J* = 8.9, 2.4 Hz, 2H), 6.48 (d, *J* = 1.9 Hz, 2H), 5.08 (s, 2H), 3.71 (ddt, *J* = 27.6, 9.3, 6.5 Hz, 4H), 1.67–1.53 (m, 4H), 1.34 (tt, *J* = 16.4, 8.3 Hz, 4H), 0.86 (t, *J* = 7.4 Hz, 6H).

*(S)-7,7′-Bis((tert-butyldimethylsilyl)oxy)-[1,1′-binaphthalene]-2,2′-diol* (**2n**): The reaction was conducted with Fe(ClO_4_)_2_ (12.6 mg, 10 mol%) and **L1** (9.8 mg, 5 mol%); 7-((*tert*-butyldimethylsilyl)oxy)naphthalen-2-ol (138.2 mg, 0.5 mmol, 1.0 equiv); and MS 4Å (153.7 mg). The desired product was obtained (103.8 mg, 76% yield) as a brown solid after purification by silica gel chromatography (PE to PE/EA = 5/1). 68:32 er (HPLC: Chiralpak IC, hexane/propan-2-ol = 97/3, 1.0 mL/min, λ = 254 nm, t*_R_* (min): major = 6.0, minor = 8.5). ^1^H-NMR (400 MHz, Chloroform-*d*) δ 7.86 (dd, *J* = 9.0, 0.7 Hz, 2H), 7.75 (d, *J* = 8.8 Hz, 2H), 7.21 (d, *J* = 8.9 Hz, 2H), 6.95 (dd, *J* = 8.8, 2.4 Hz, 2H), 6.46 (d, *J* = 2.4 Hz, 2H), 5.07 (s, 2H), 0.83 (s, 18H), −0.03 (s, 6H), −0.06 (s, 6H) (Figure S17); ^13^C-NMR (100 MHz, Chloroform-*d*) δ 155.3, 153.2, 134.9, 131.1, 129.9, 125.1, 119.9, 115.3, 111.7, 109.8, 25.8, 18.3, −4.5 (Figure S18); HRMS (ESI^−^), *m/z* calc′d for C_32_H_41_O_4_Si_2_ [M − H]^−^: 545.2549, found 545.2578; ${\left[\alpha \right]}_{D}^{24}$ = 56.6 (*c* = 1.0, CHCl_3_); M.p. 118–122 °C.

*(S)-7,7′-Dimethoxy-[1,1′-binaphthalene]-2,2′-diol* (**2o**) [78]: The reaction was conducted with Fe(ClO_4_)_2_ (12.4 mg, 10 mol%) and **L1** (9.4 mg, 5 mol%); 7-methoxynaphthalen-2-ol (87.3 mg, 0.5 mmol, 1.0 equiv); and MS 4Å (156.3 mg). The desired product was obtained (56.3 mg, 65% yield) as a white solid after purification by silica gel chromatography (PE to PE/EA = 5/1). 59:41 er (HPLC: Chiralcel OD-H, hexane/propan-2-ol = 85/15, 1.0 mL/min, λ = 230 nm, t*_R_* (min): major = 11.1, minor = 18.8). ^1^H-NMR (400 MHz, Chloroform-*d*) δ 7.88 (dd, *J* = 9.0, 0.7 Hz, 2H), 7.78 (d, *J* = 8.9 Hz, 2H), 7.22 (d, *J* = 8.8 Hz, 2H), 7.03 (dd, *J* = 8.9, 2.5 Hz, 2H), 6.48 (d, *J* = 2.5 Hz, 2H), 3.57 (s, 6H).

## 4. Conclusions

In summary, we have developed an iron/bisquinolyldiamine-catalyzed asymmetric oxidative coupling of 2-naphthols. This method employs in situ-formed iron complexes from Fe(ClO_4_)_2_ and readily available ligand **L1** and uses 1 atm oxygen as the oxidant. The atom economy of this transformation, the easily available catalyst, and operationally simple procedure provide new applications of asymmetric iron catalysis. Further studies on synthesizing a library of nitrogen ligands and extending their applications are underway in our laboratory.

## Figures and Tables

**Table 1:**
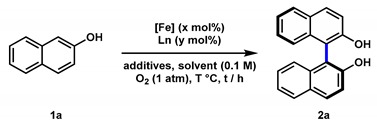
Conditions Screening *^a.^*

Entry	Fe (x mol%)	Ln (y mol%)	Additives	Solvent	T °C	t/h	conv. (%) *^b^*	er (%) *^c^*
1	Fe(ClO_4_)_2_ (5.0)	**L1**(5.0)	-	MeOH	30	28.5	-	n.d. *^f^*
2	Fe(ClO_4_)_2_ (5.0)	**L1** (5.0)	-	DCE	30	28.5	57 *^d^*	73:27
3	Fe(ClO_4_)_2_ (5.0)	**L1** (5.0)	-	CHCl_3_	30	28.5	68 *^d^*	77:23
4	Fe(ClO_4_)_2_ (5.0)	**L1** (5.0)	-	toluene	30	28.5	22 *^d^*	80:20
5	Fe(ClO_4_)_2_ (5.0)	**L1** (5.0)	-	PhCl	30	28.5	53 *^d^*	79:21
6	Fe(ClO_4_)_2_ (5.0)	**L1** (5.0)	MS 4Å	PhCl	30	5.0	12 *^d^*	80:20
7	Fe(ClO_4_)_2_ (5.0)	**L1** (5.0)	MS 4Å	PhCl	50	5.0	39 *^d^*	78:22
8	Fe(ClO_4_)_2_ (5.0)	**L1** (5.0)	MS 4Å	PhCl	70	5.0	38 *^d^*	73:27
9	Fe(ClO_4_)_2_ (5.0)	**L1** (5.0)	MS 4Å	PhCl	90	5.0	28 *^d^*	70:30
10	Fe(ClO_4_)_3_ (5.0)	**L1** (5.0)	MS 4Å	PhCl	50	5.0	76	76:24
11	Fe(OAc)_2_ (5.0)	**L1** (5.0)	MS 4Å	PhCl	50	5.0	16	n.d. *^f^*
12	Fe(OTf)_2_ (5.0)	**L1** (5.0)	MS 4Å	PhCl	50	5.0	19	n.d. *^f^*
13	Fe(acac)_2_ (5.0)	**L1** (5.0)	MS 4Å	PhCl	50	5.0	23	n.d. *^f^*
14	FeCl_2_ (5.0)	**L1** (5.0)	MS 4Å	PhCl	50	5.0	20	n.d. *^f^*
15	Fe(ClO_4_)_2_ (7.5)	**L1** (5.0)	MS 4Å	PhCl	50	5.0	78	77:23
16	Fe(ClO_4_)_2_ (10.0)	L1 (5.0)	MS 4Å	PhCl	50	5.0	88(84 *^e^*)	80:20
17	Fe(ClO_4_)_2_ (12.5)	**L1** (5.0)	MS 4Å	PhCl	50	5.0	95	72:28
18	Fe(ClO_4_)_2_ (5.0)	**L1** (10.0)	MS 4Å	PhCl	50	5.0	39	77:23
19	Fe(ClO_4_)_2_ (10.0)	**L1** (10.0)	MS 4Å	PhCl	50	5.0	86	77:23
20	Fe(ClO_4_)_2_ (10.0)	**L2** (5.0)	MS 4Å	PhCl	50	5.0	90	70:30
21	Fe(ClO_4_)_2_ (10.0)	**L3** (5.0)	MS 4Å	PhCl	50	5.0	85	60:40
22	Fe(ClO_4_)_2_ (10.0)	**L4** (5.0)	MS 4Å	PhCl	50	5.0	53 *^e^*	78:22
23	Fe(ClO_4_)_2_ (10.0)	**L5** (5.0)	MS 4Å	PhCl	50	5.0	67 *^e^*	55:45
24	Fe(ClO_4_)_2_ (10.0)	**L6** (5.0)	MS 4Å	PhCl	50	5.0	-	n.d. *^f^*
25	Fe(ClO_4_)_2_ (10.0)	**L7** (5.0)	MS 4Å	PhCl	50	5.0	-	n.d. *^f^*
26	Fe(ClO_4_)_2_ (10.0)	**L8** (5.0)	MS 4Å	PhCl	50	5.0	-	n.d. *^f^*

*^a^* All reactions carried out with **1a** (0.5 mmol), MS 4Å (150 mg) in 5 mL solvent under O_2_ atmosphere (1 atm). *^b^* Conversions determined by GC using dodecane as an internal standard. *^c^* Determined by HPLC (Chiralpak AS-H). *^d^* Determined by ^1^H-NMR analysis. *^e^* Isolated yield. *^f^* n.d.: not detected.

## Supplementary Materials

The following are available online. NMR and HPLC spectra.
