# Supplementary material for: Enantioselective Iron/Bisquinolyldiamine Ligand-Catalyzed Oxidative Coupling Reaction of 2-Naphthols
Source: Molecules. 2020 Feb 14;25(4):852. doi: 10.3390/molecules25040852 (PMC7070846; doi:10.3390/molecules25040852)
Supplement: Supplementary file 1 [file molecules-25-00852-s001.pdf]

Supplementary Materials for

Enantioselective Iron/Bisquinolyldiamine Ligand-Catalyzed Oxidative Coupling Reaction of 2-Naphthols

Lin-Yang Wu, Muhammad Usman and Wen-Bo Liu*

Sauvage Center for Molecular Sciences; Engineering Research Center of Organosilicon Compounds & Materials, Ministry of Education; College of Chemistry and Molecular Sciences; Wuhan University, Wuhan, Hubei 430072, China

Contents

Copies of NMR Spectra **S2**

Copies of HPLC Spectrum **S19**

Copies of NMR Spectra

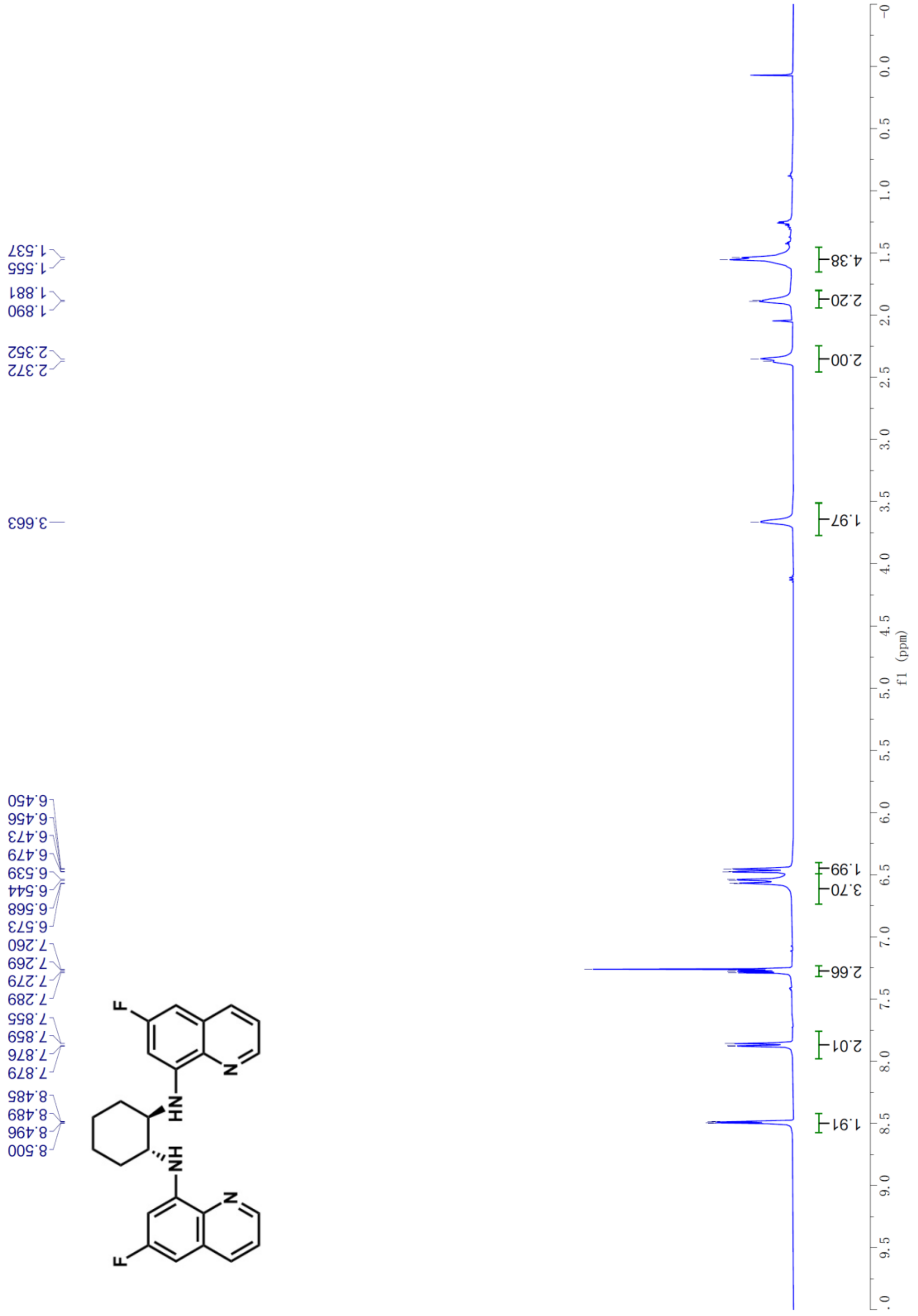

^{1}H NMR of **L2**

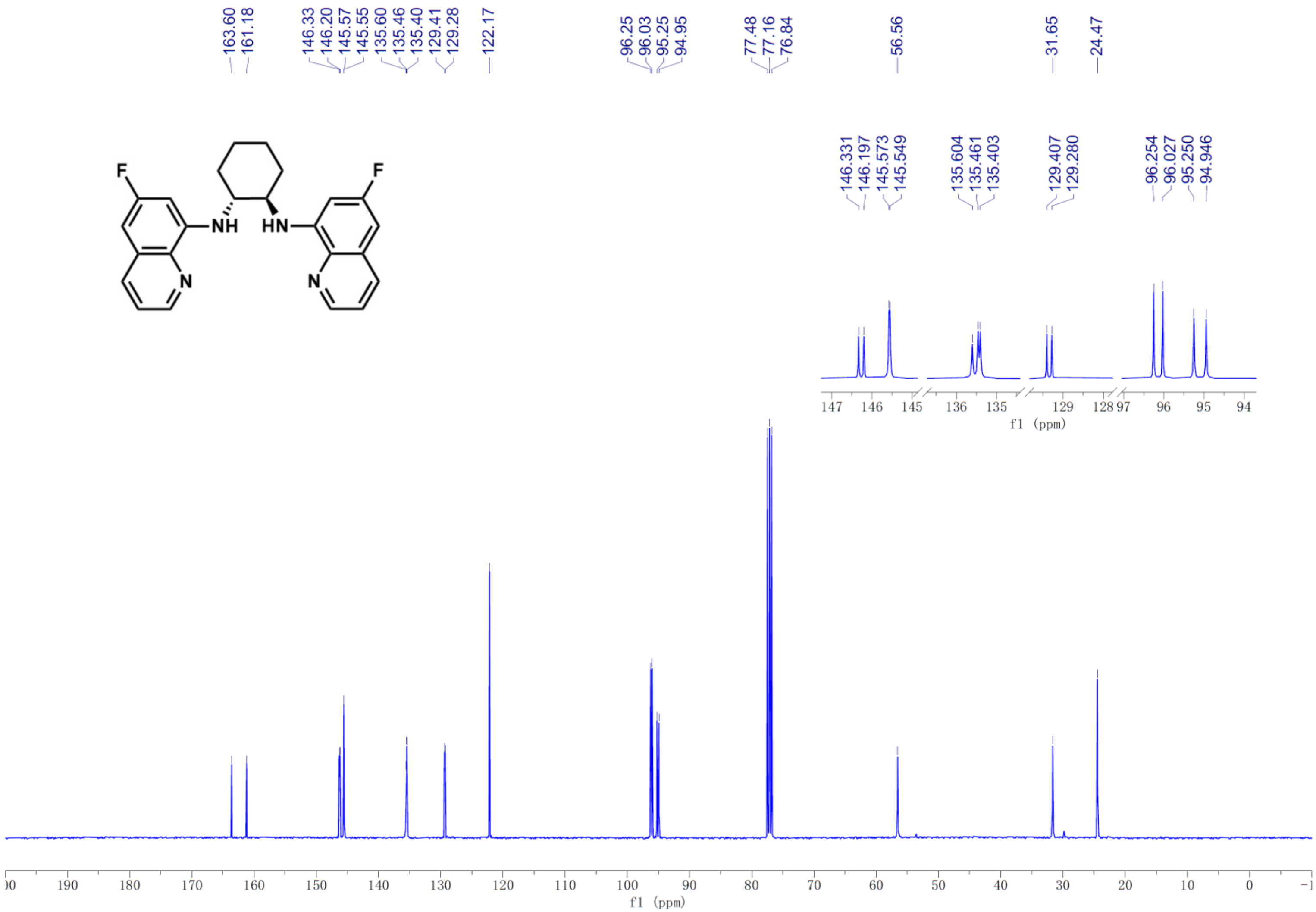
163.60
161.18
146.33
146.20
145.57
145.55
135.60
135.46
135.40
129.41
129.28
122.17
96.25
96.03
95.25
94.95
77.48
77.16
76.84
56.56
31.65
24.47
146.331
146.197
145.573
145.549
135.604
135.461
135.403
129.407
129.280
96.254
96.027
95.250
94.946
f1 (ppm)
F
NH
HN
N

^{13}C NMR of **L2**

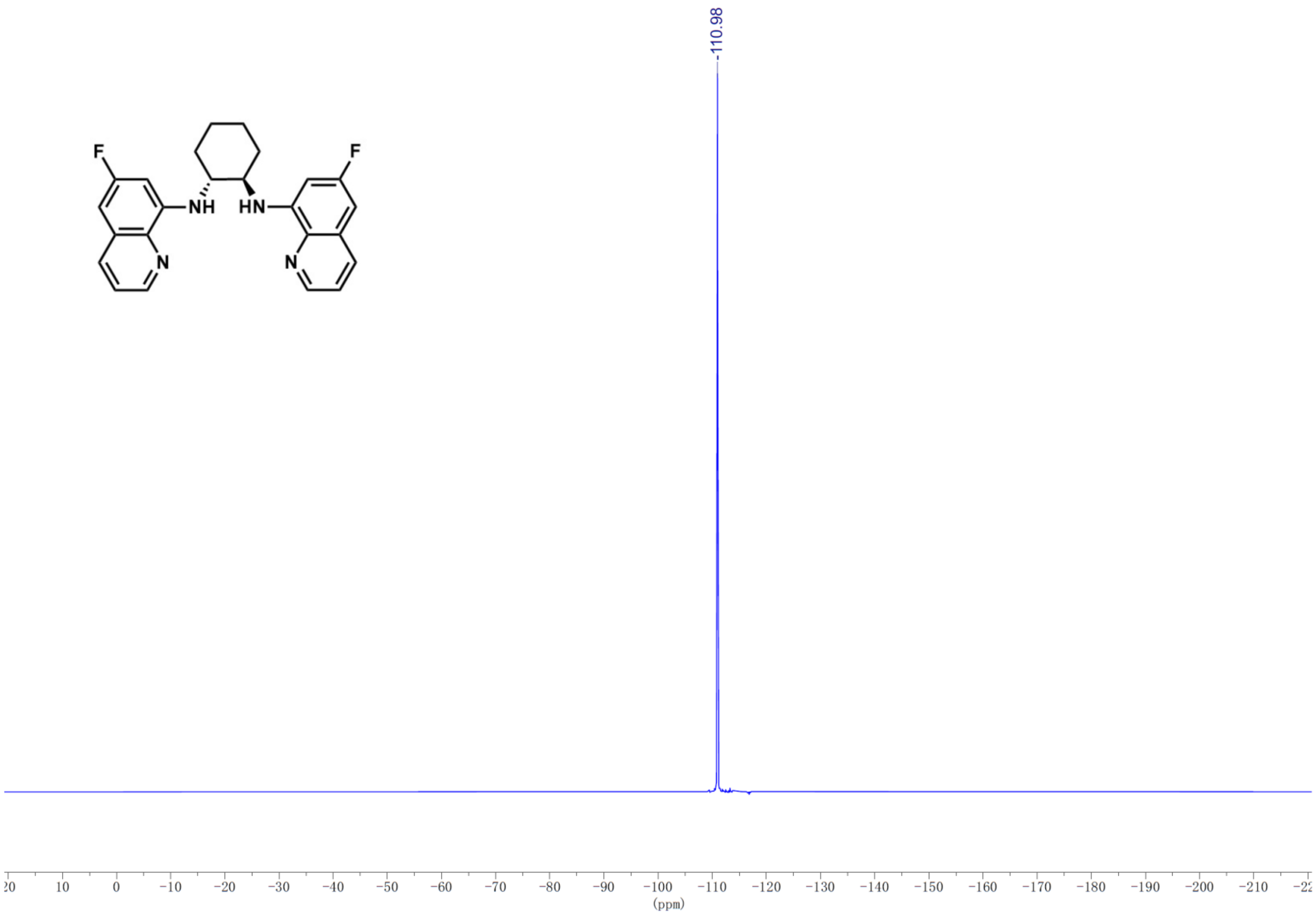

F
NH
HN
F
N
N
-110.98
20
10
0
−10
−20
−30
−40
−50
−60
−70
−80
−90
−100
−110
−120
−130
−140
−150
−160
−170
−180
−190
−200
−210
(ppm)

^{19}F NMR of **L2**

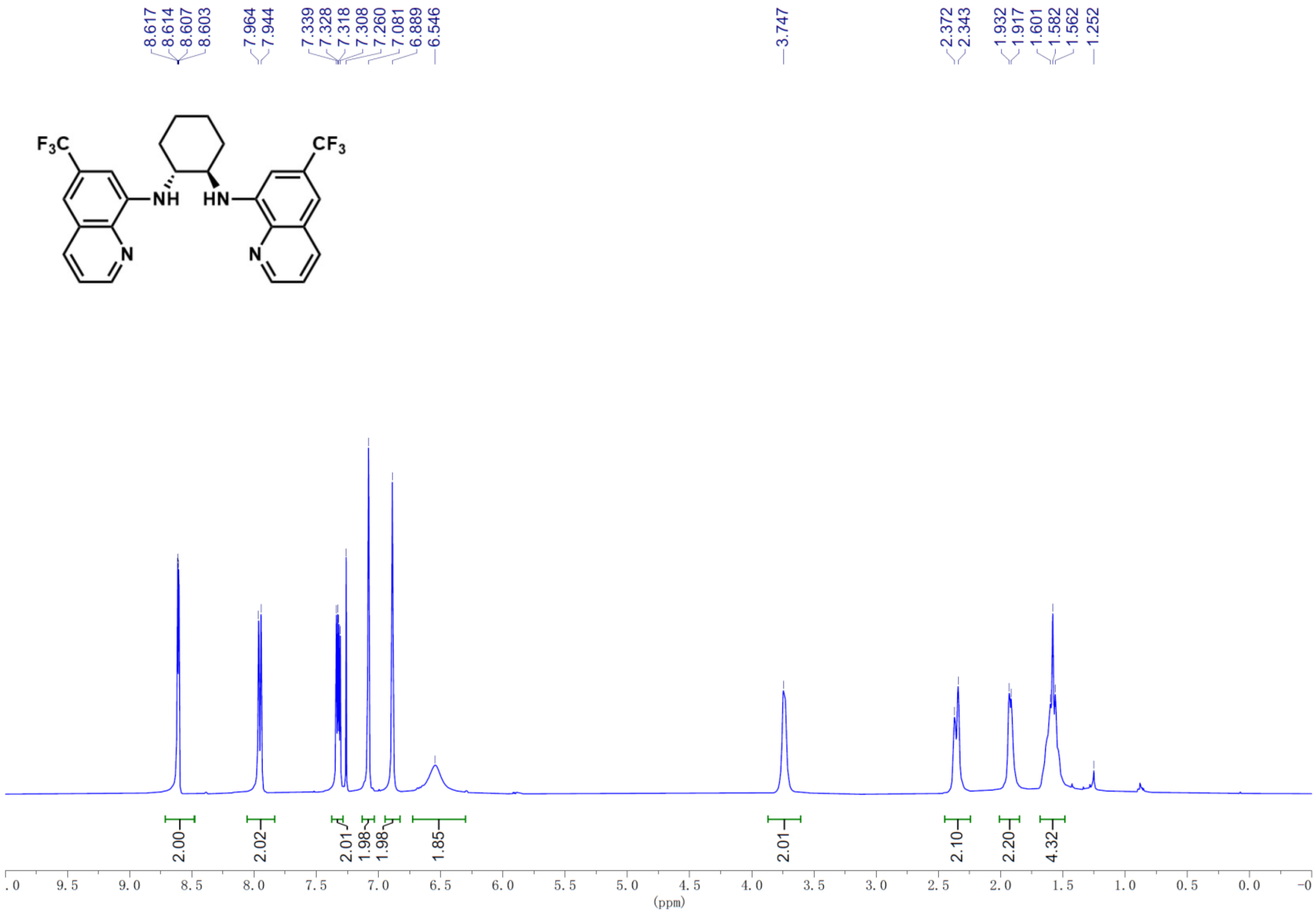
8.617
8.614
8.607
8.603
7.964
7.944
7.339
7.328
7.318
7.308
7.260
7.081
6.889
6.546
3.747
2.372
2.343
1.932
1.917
1.601
1.582
1.562
1.252
F_3C
CF_3
NH
HN
N
N
2.00
2.02
2.01
1.98
1.98
1.85
2.01
2.10
2.20
4.32
(ppm)

^{1}H NMR of **L3**

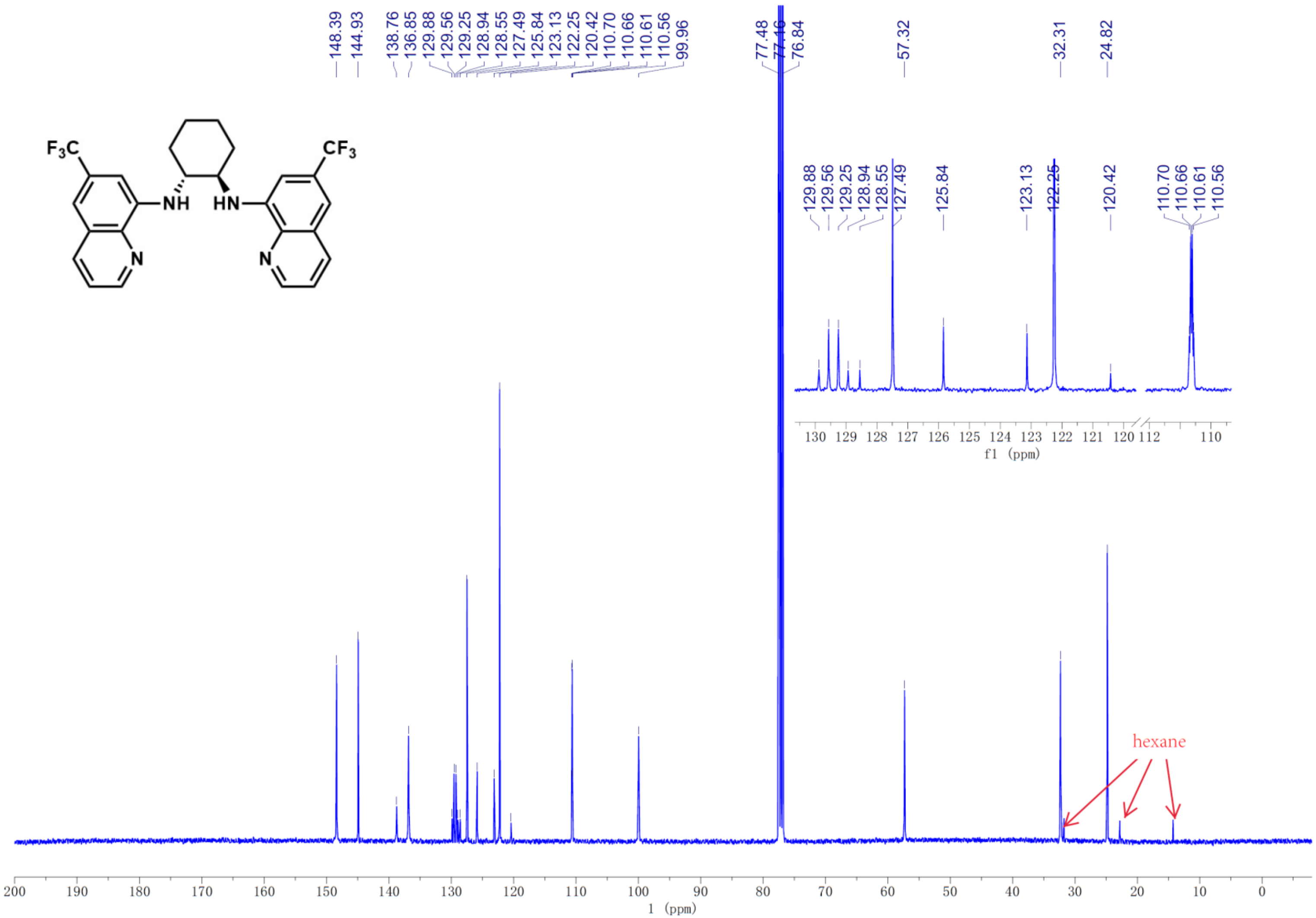
148.39
144.93
138.76
136.85
129.88
129.56
129.25
128.94
128.55
127.49
125.84
123.13
122.25
120.42
110.70
110.66
110.61
110.56
99.96
77.48
77.16
76.84
57.32
32.31
24.82
hexane
1 (ppm)
f1 (ppm)
F3C
CF3
NH
HN
N

^{13}C NMR of **L3**

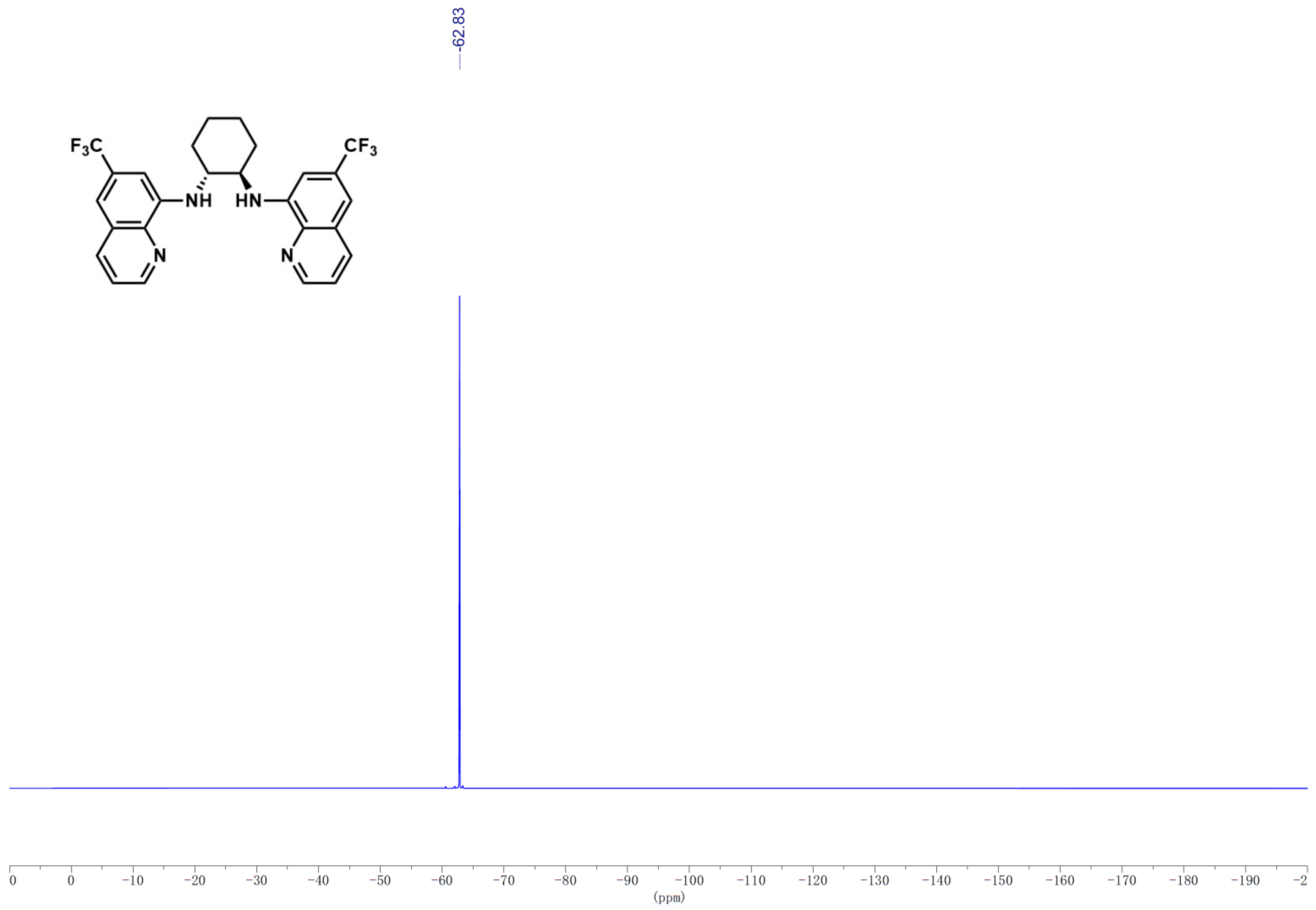

-62.83
F3C
CF3
NH
HN
N
N
0
0
−10
−20
−30
−40
−50
−60
−70
−80
−90
−100
(ppm)
−110
−120
−130
−140
−150
−160
−170
−180
−190
−2

^{19}F NMR of **L3**

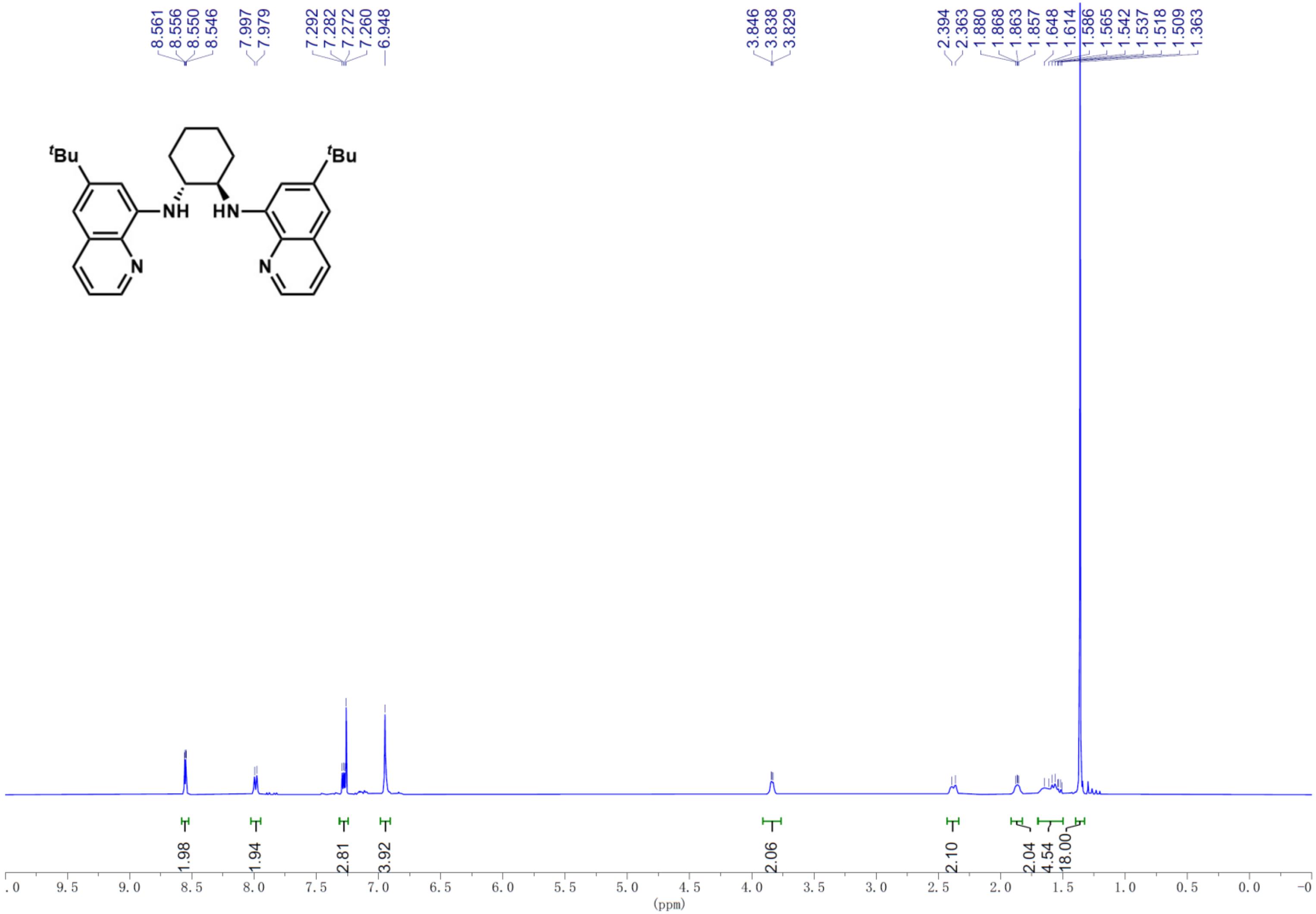
8.561
8.556
8.550
8.546
7.997
7.979
7.292
7.282
7.272
7.260
6.948
3.846
3.838
3.829
2.394
2.363
1.880
1.868
1.863
1.857
1.648
1.614
1.586
1.565
1.542
1.537
1.518
1.509
1.363
tBu
NH
HN
N
1.98
1.94
2.81
3.92
2.06
2.10
2.04
4.54
18.00
(ppm)

^{1}H NMR of **L4**

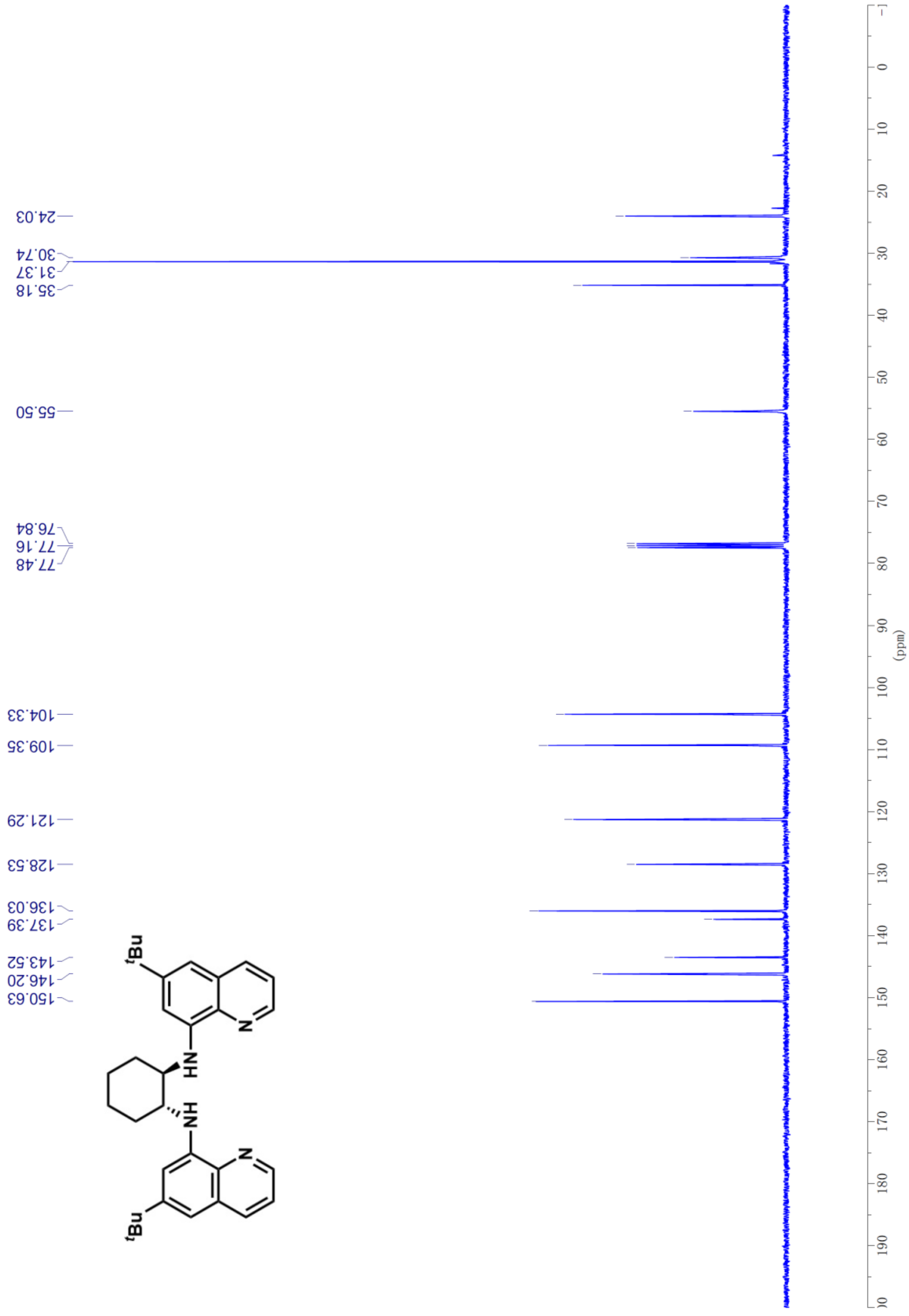
24.03
30.74
31.37
35.18
55.50
76.84
77.16
77.48
104.33
109.35
121.29
128.53
136.03
137.39
143.52
146.20
150.63
tBu
HN
NH
N
(ppm)
0
10
20
30
40
50
60
70
80
90
100
110
120
130
140
150
160
170
180
190

^{13}C NMR of **L4**

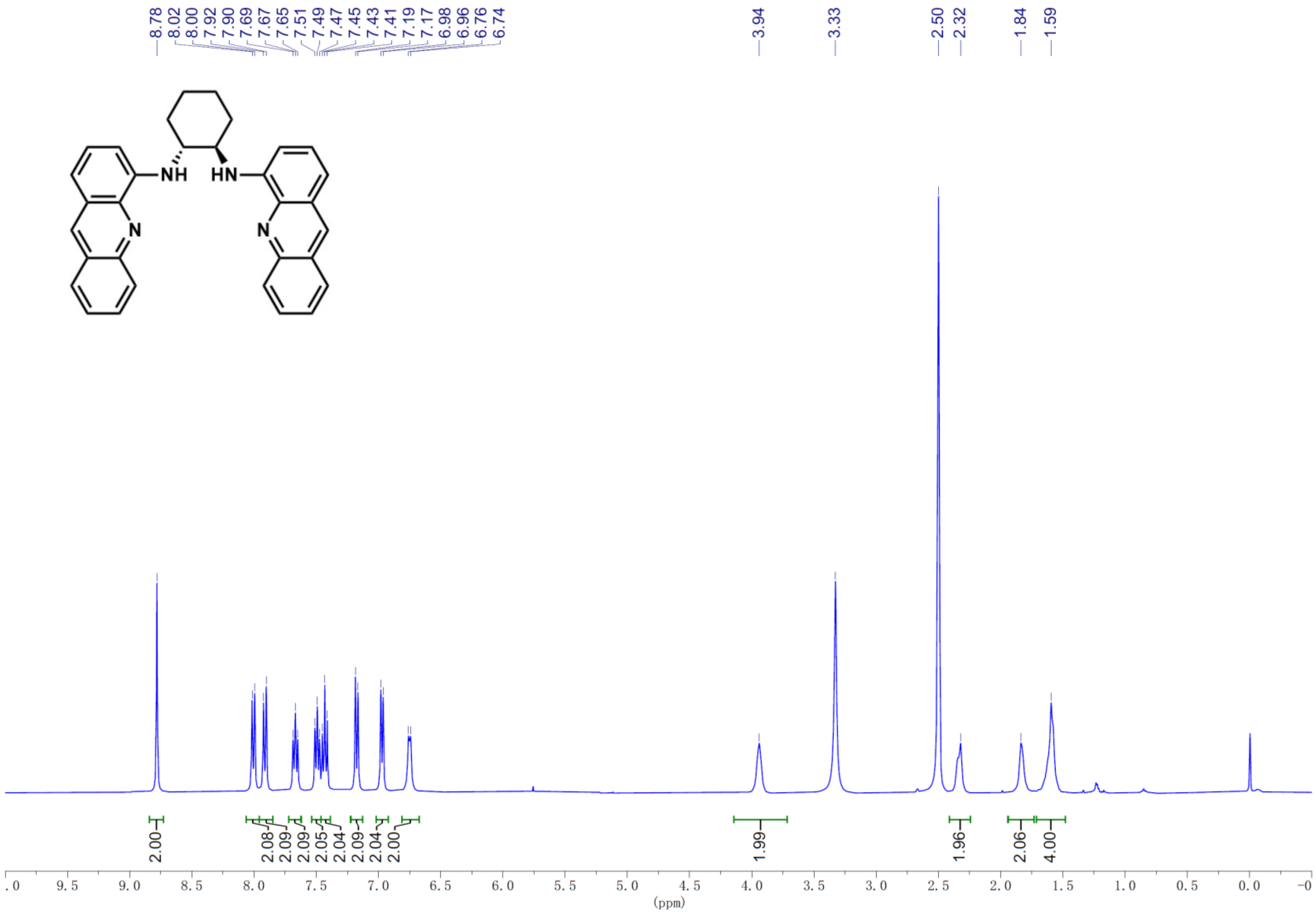
8.78
8.02
8.00
7.92
7.90
7.69
7.67
7.65
7.51
7.49
7.47
7.45
7.43
7.41
7.19
7.17
6.98
6.96
6.76
6.74
3.94
3.33
2.50
2.32
1.84
1.59
NH
HN
N
N
2.00
2.08
2.09
2.09
2.05
2.04
2.09
2.04
2.00
1.99
1.96
2.06
4.00
(ppm)

^{1}H NMR of **L5**

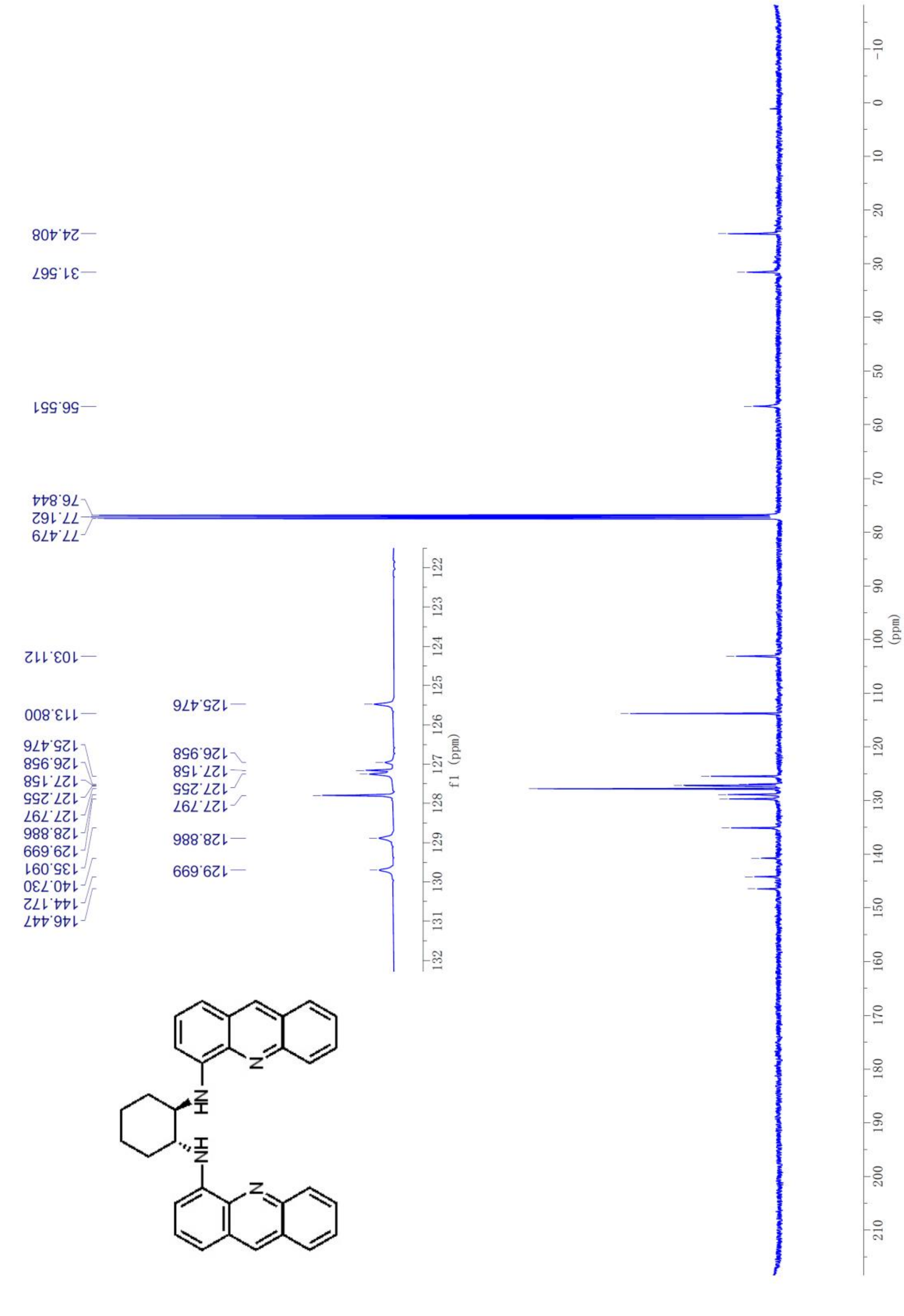
24.408
31.567
56.551
76.844
77.162
77.479
103.112
113.800
125.476
126.958
127.158
127.255
127.797
128.886
129.699
135.091
140.730
144.172
146.447
125.476
126.958
127.158
127.255
127.797
128.886
129.699
f1 (ppm)
(ppm)
NH
HN
N
N

^{13}C NMR of **L5**

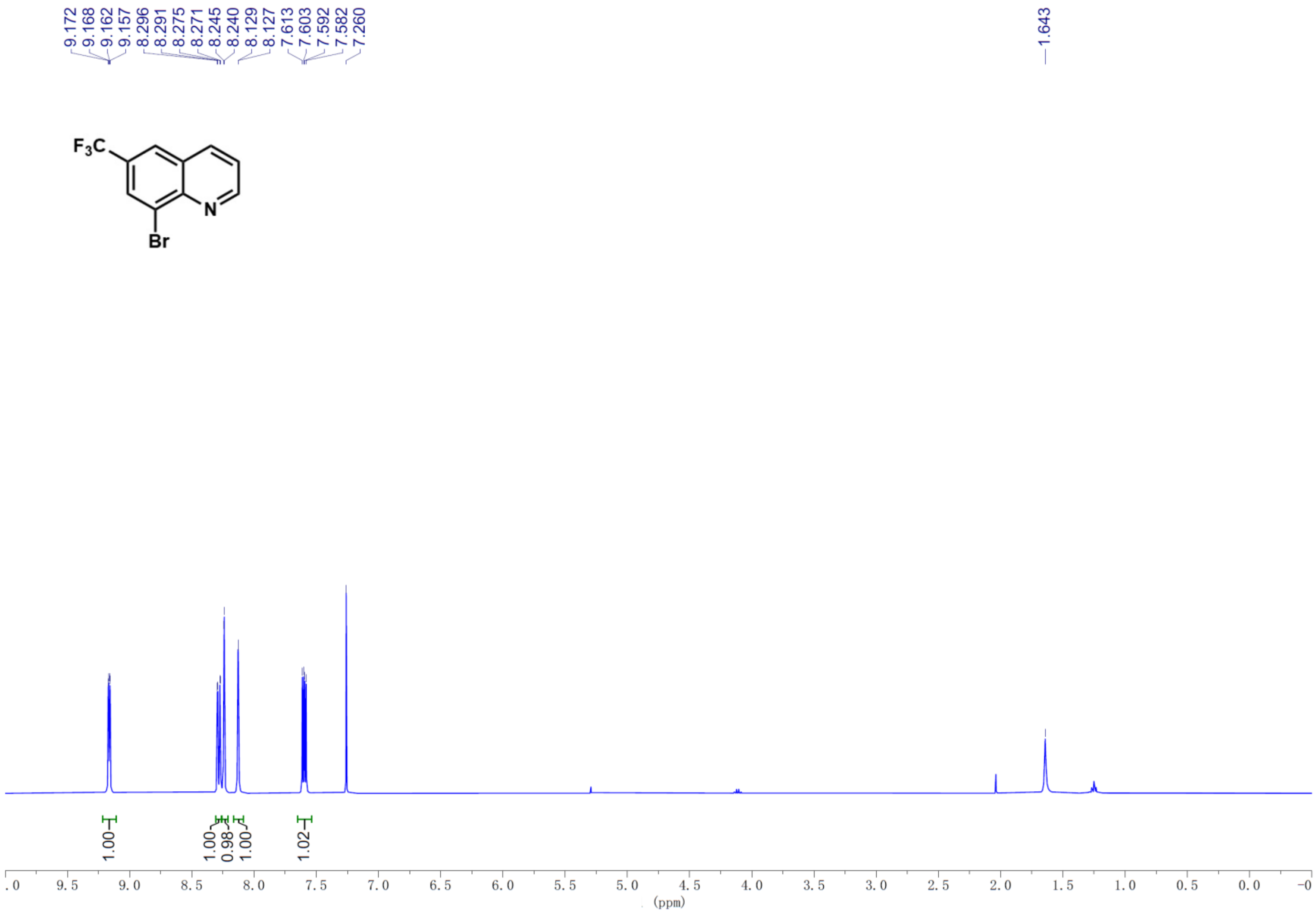

9.172
9.168
9.162
9.157
8.296
8.291
8.275
8.271
8.245
8.240
8.129
8.127
7.613
7.603
7.592
7.582
7.260
1.643
F3C
N
Br
1.00
1.00
0.98
1.00
1.02
9.5
9.0
8.5
8.0
7.5
7.0
6.5
6.0
5.5
5.0
4.5
4.0
3.5
3.0
2.5
2.0
1.5
1.0
0.5
0.0
(ppm)

^{1}H NMR of **4c**

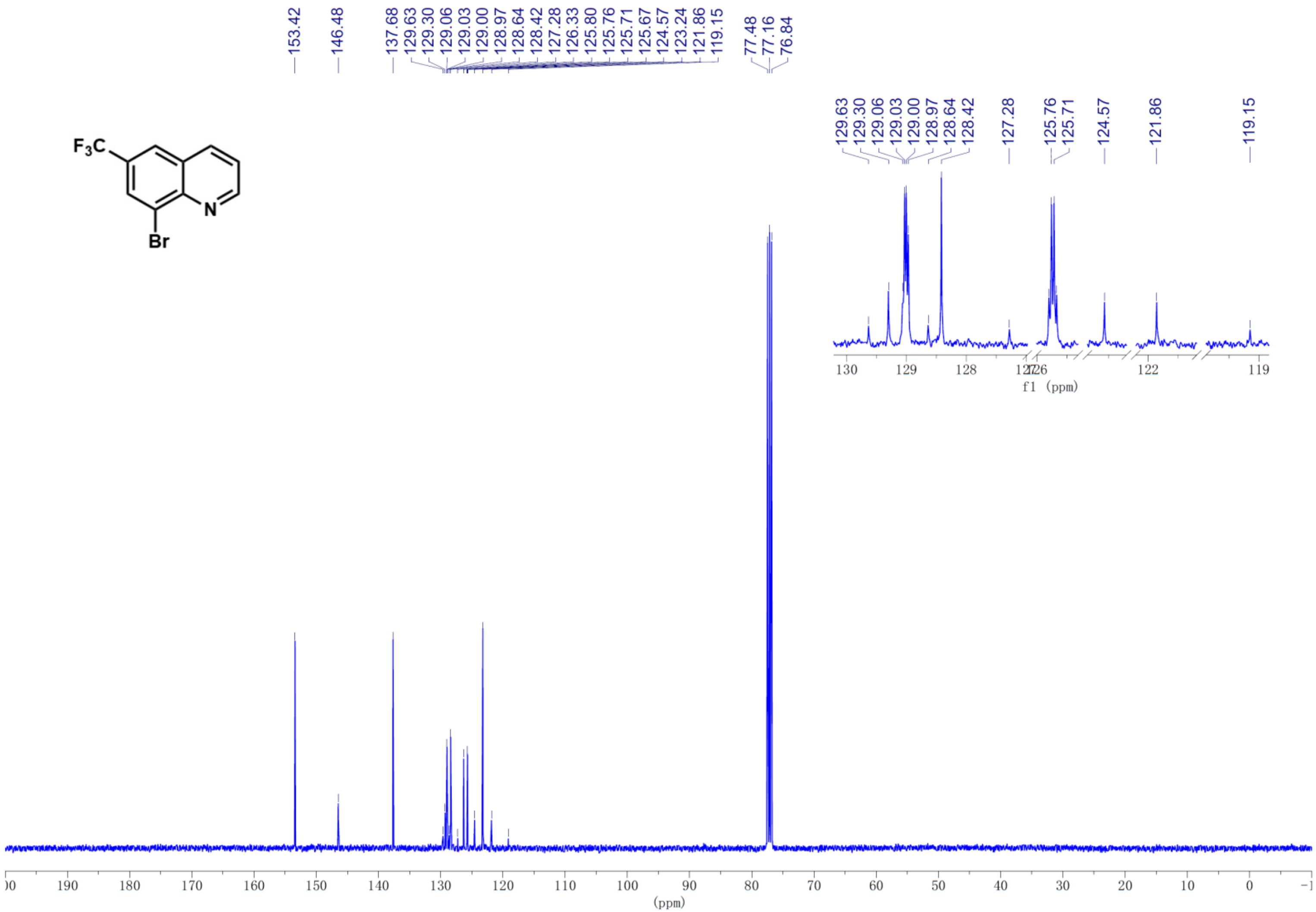
153.42
146.48
137.68
129.63
129.30
129.06
129.03
129.00
128.97
128.64
128.42
127.28
126.33
125.80
125.76
125.71
125.67
124.57
123.24
121.86
119.15
77.48
77.16
76.84
F3C
N
Br
f1 (ppm)
(ppm)

^{13}C NMR of **4c**

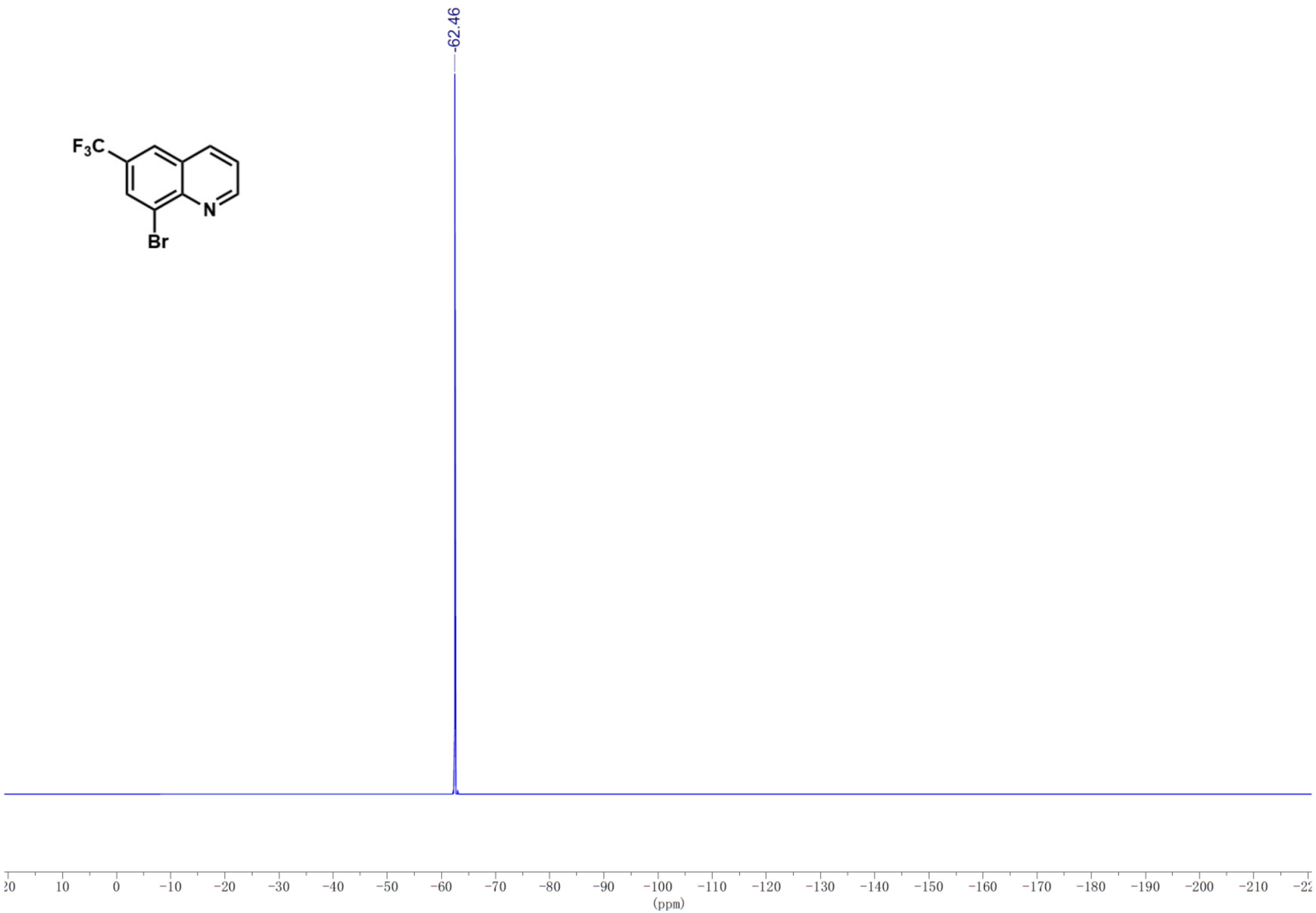

F3C
N
Br
-62.46
20
10
0
−10
−20
−30
−40
−50
−60
−70
−80
−90
−100
(ppm)
−110
−120
−130
−140
−150
−160
−170
−180
−190
−200
−210

^{19}F NMR of **4c**

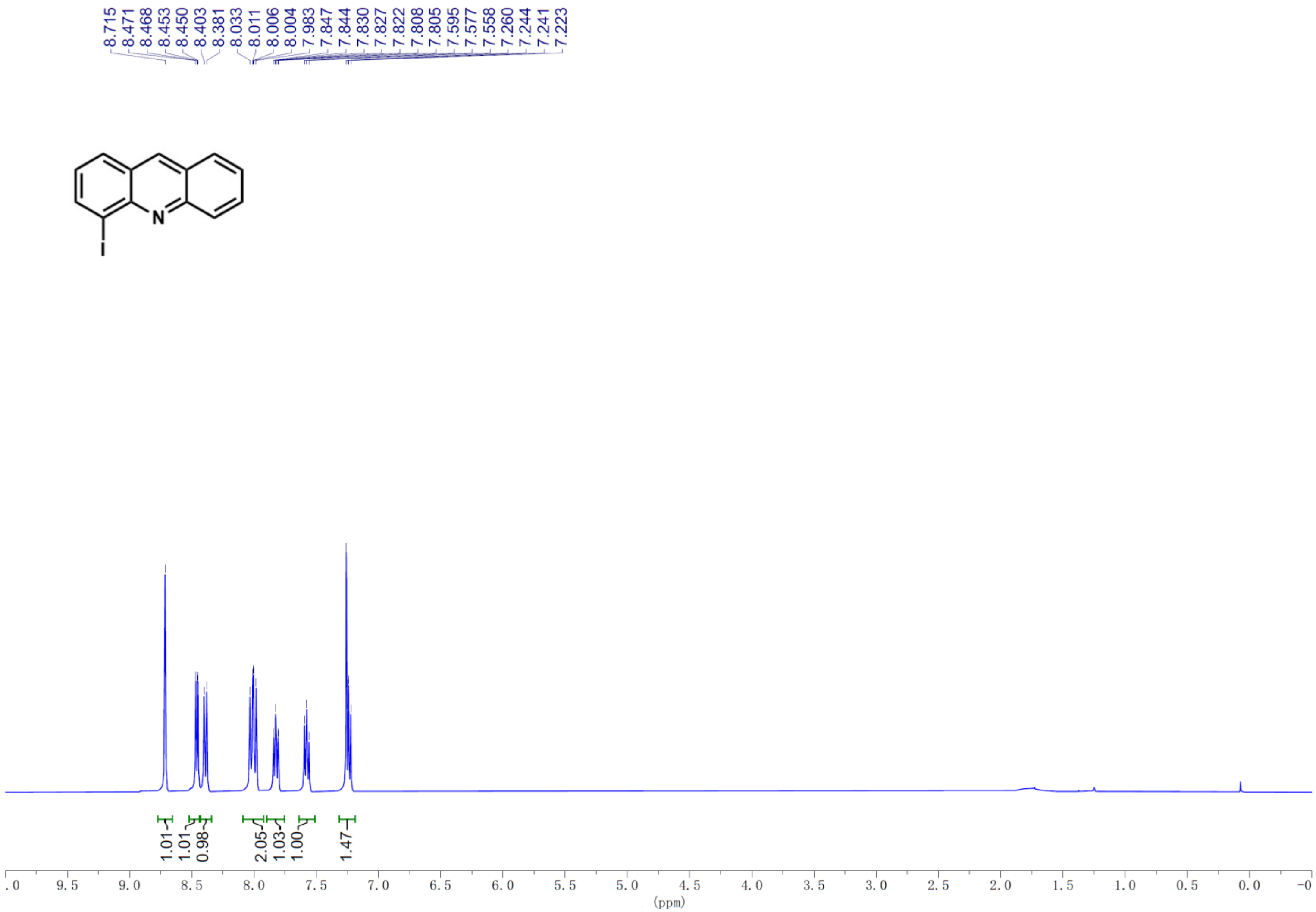
8.715
8.471
8.468
8.453
8.450
8.403
8.381
8.033
8.011
8.006
8.004
7.983
7.847
7.844
7.830
7.827
7.822
7.808
7.805
7.595
7.577
7.558
7.260
7.244
7.241
7.223
N
I
1.01
1.01
0.98
2.05
1.03
1.00
1.47
(ppm)

^{1}H NMR of **4e**

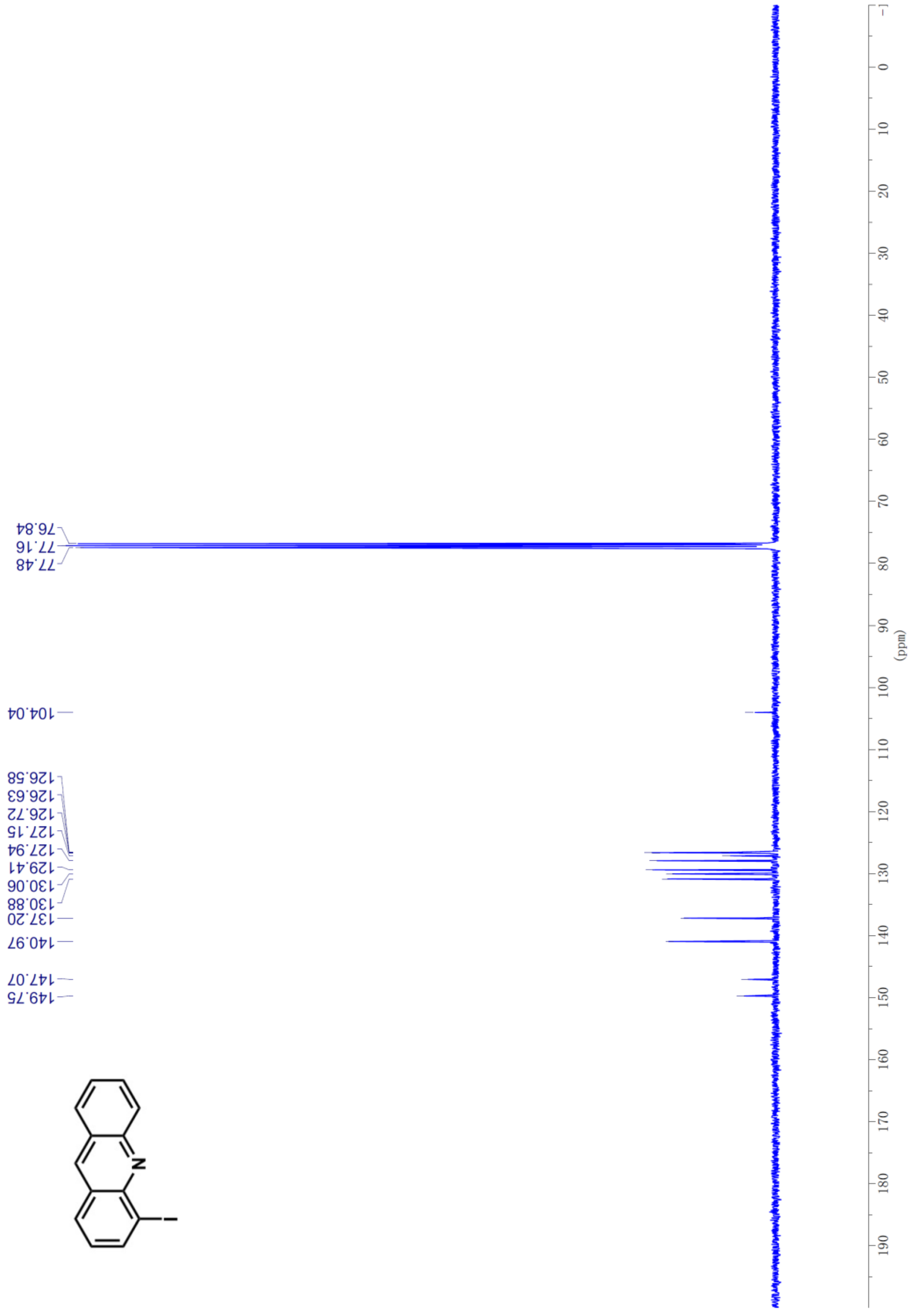
76.84
77.16
77.48
104.04
126.58
126.63
126.72
127.15
127.94
129.41
130.06
130.88
137.20
140.97
147.07
149.75
N
(ppm)

^{13}C NMR of **4e**

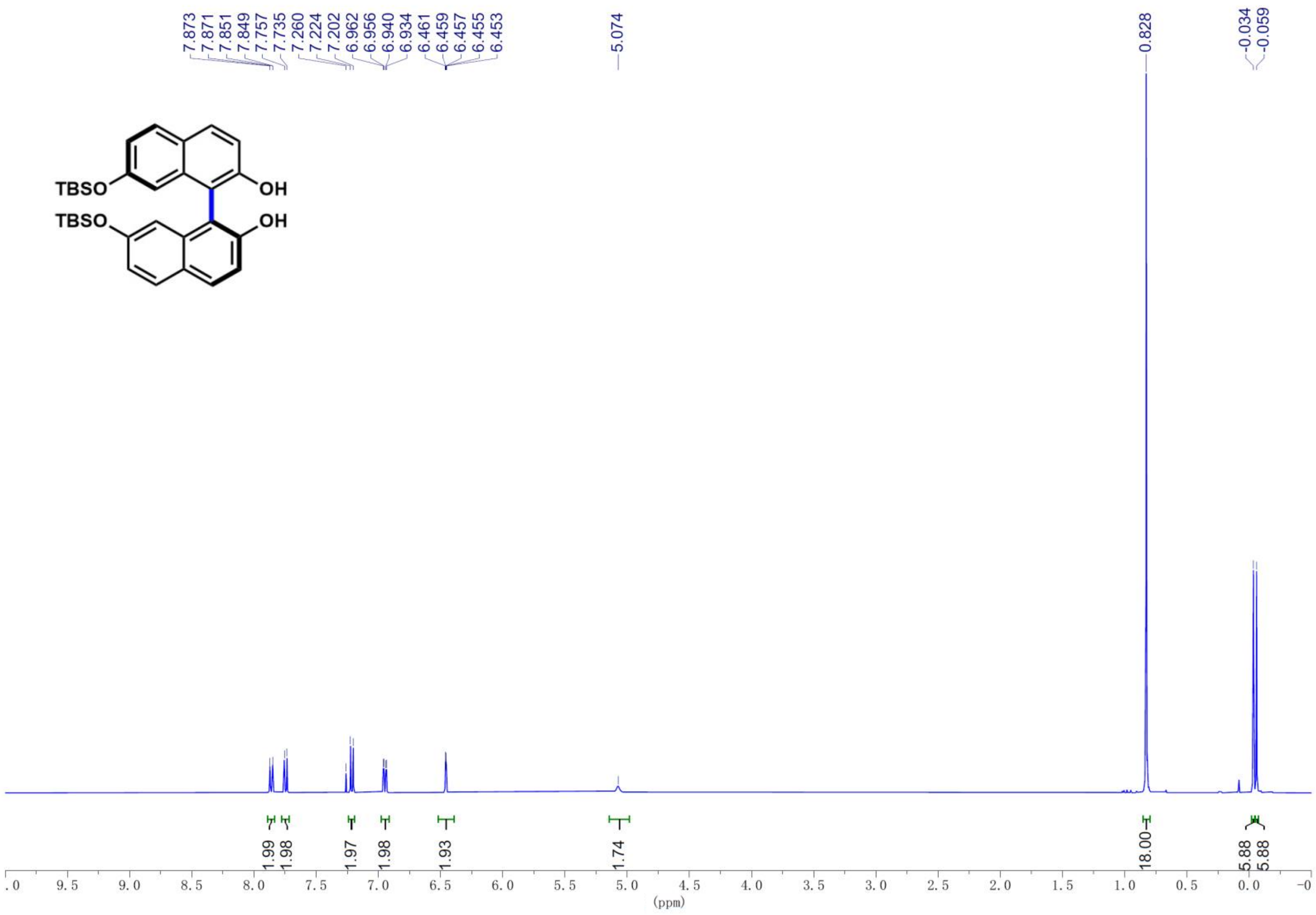
7.873
7.871
7.851
7.849
7.757
7.735
7.260
7.224
7.202
6.962
6.956
6.940
6.934
6.461
6.459
6.457
6.455
6.453
5.074
0.828
-0.034
-0.059
TBSO
OH
TBSO
OH
1.99
1.98
1.97
1.98
1.93
1.74
18.00
5.88
5.88
(ppm)

^{1}H NMR of **2n**

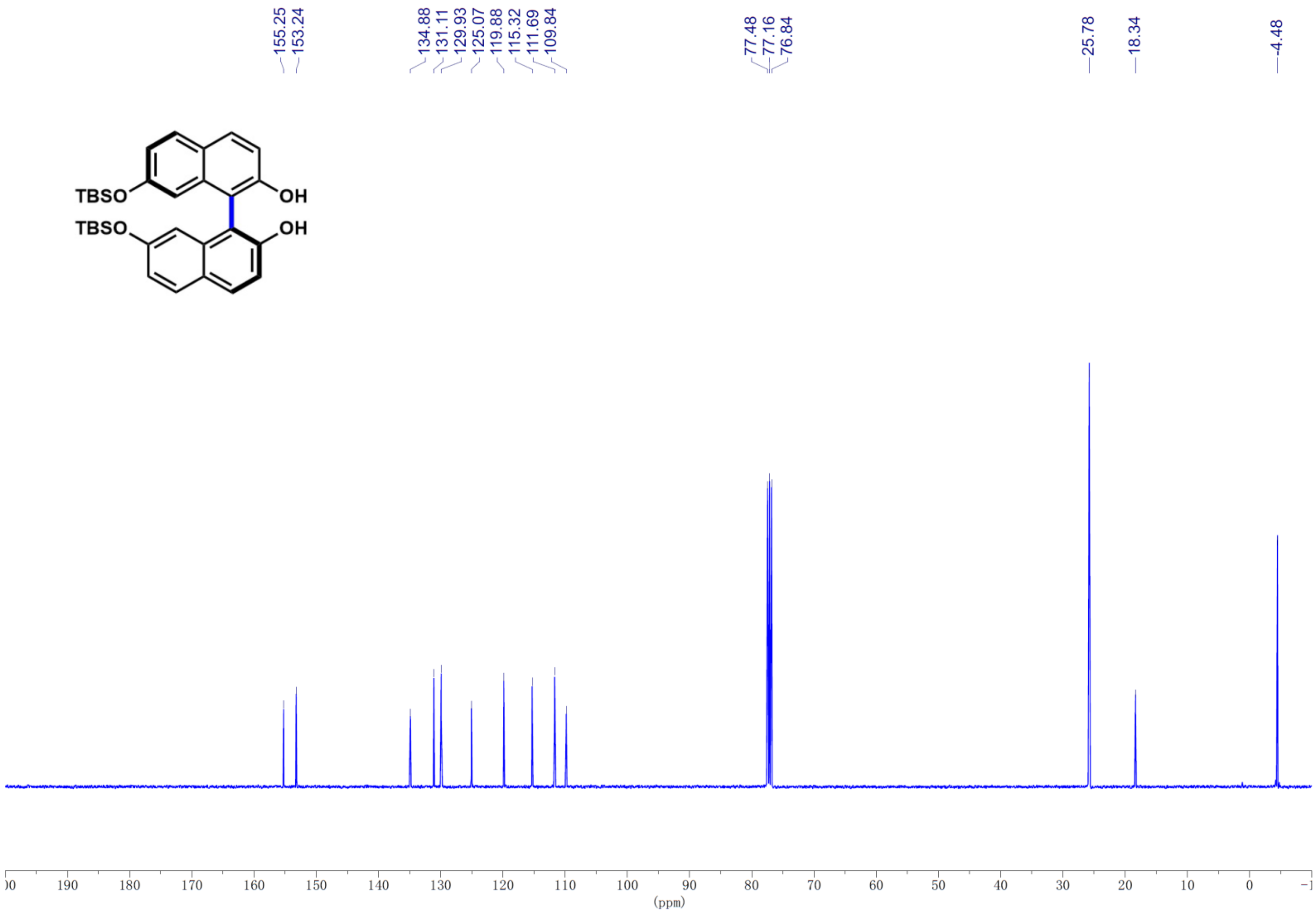

155.25
153.24
134.88
131.11
129.93
125.07
119.88
115.32
111.69
109.84
77.48
77.16
76.84
25.78
18.34
-4.48
TBSO
TBSO
OH
OH
(ppm)

^{13}C NMR of **2n**

Copies of HPLC Spectrum

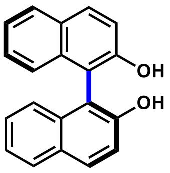

Additional Info : Peak(s) manually integrated

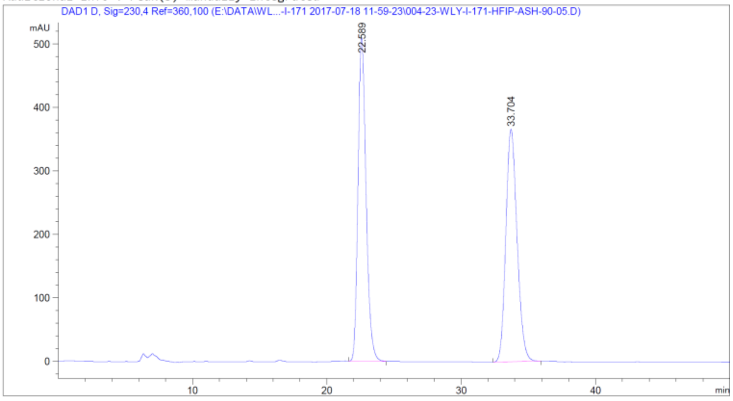

Area Percent Report

Sorted By : Signal
Multiplier : 1.0000
Dilution : 1.0000
Use Multiplier & Dilution Factor with ISTDs

Signal 1: DAD1 D, Sig=230,4 Ref=360,100

Peak #	RetTime [min]	Type	Width [min]	Area [mAU*s]	Height [mAU]	Area %
1	22.589	BB	0.6379	2.13227e4	508.31418	50.0704
2	33.704	BB	0.8048	2.12627e4	366.27551	49.9296

Additional Info : Peak(s) manually integrated

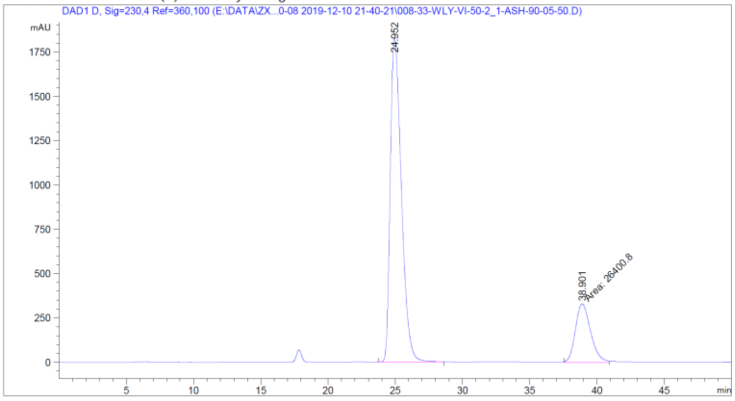

Area Percent Report

Sorted By : Signal
Multiplier : 1.0000
Dilution : 1.0000
Use Multiplier & Dilution Factor with ISTDs

Signal 1: DAD1 D, Sig=230,4 Ref=360,100

Peak #	RetTime [min]	Type	Width [min]	Area [mAU*s]	Height [mAU]	Area %
1	24.952	BB	0.6798	1.04650e5	1825.58789	79.8546
2	38.901	MM	1.3212	2.64008e4	333.04184	20.1454

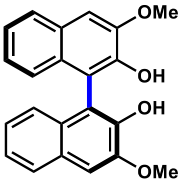

Additional Info : Peak(s) manually integrated

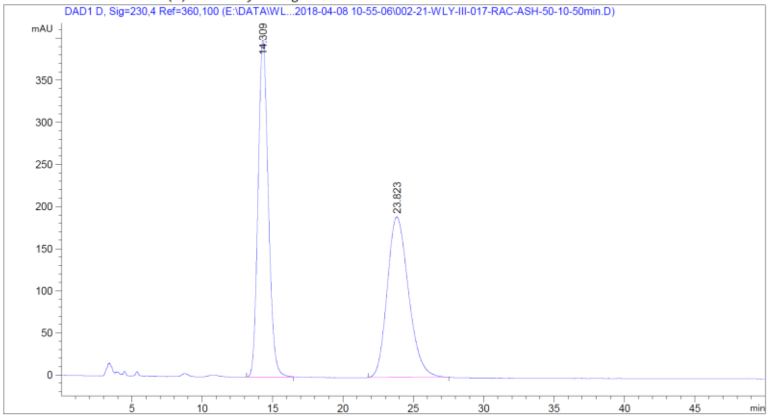

===
Area Percent Report
===

Sorted By : Signal
Multiplier : 1.0000
Dilution : 1.0000
Use Multiplier & Dilution Factor with ISTDs

Signal 1: DAD1 D, Sig=230,4 Ref=360,100

Peak #	RetTime [min]	Type	Width [min]	Area [mAU*s]	Height [mAU]	Area %
1	14.309	BB	0.7439	2.02275e4	400.07605	50.2202
2	23.823	BB	1.2303	2.00501e4	190.74997	49.7798

Additional Info : Peak(s) manually integrated

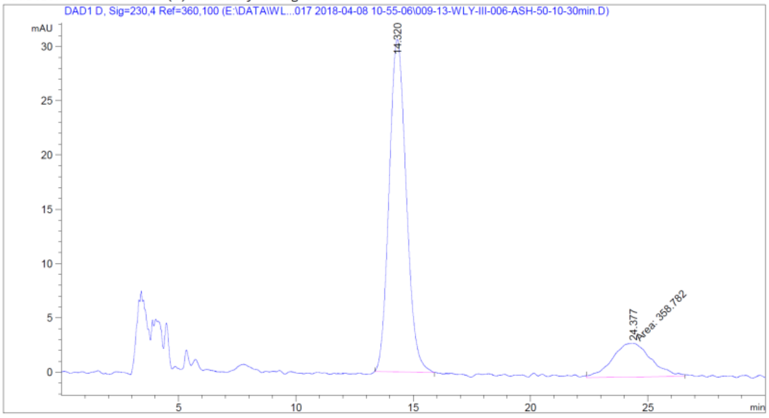

===
Area Percent Report
===

Sorted By : Signal
Multiplier : 1.0000
Dilution : 1.0000
Use Multiplier & Dilution Factor with ISTDs

Signal 1: DAD1 D, Sig=230,4 Ref=360,100

Peak #	RetTime [min]	Type	Width [min]	Area [mAU*s]	Height [mAU]	Area %
1	14.320	BB	0.5945	1552.13379	30.61634	81.2246
2	24.377	MM	1.9152	358.78198	3.12225	18.7754

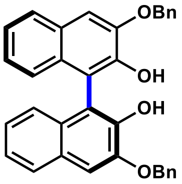

Additional Info : Peak(s) manually integrated

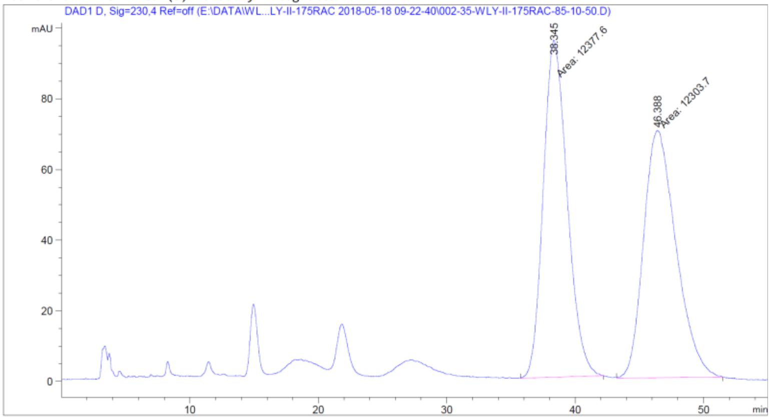


```
=====================================================================
                        Area Percent Report
=====================================================================

Sorted By             :      Signal
Multiplier            :      1.0000
Dilution              :      1.0000
Use Multiplier & Dilution Factor with ISTDs


Signal 1: DAD1 D, Sig=230,4 Ref=off

Peak RetTime Type  Width     Area      Height     Area
  #   [min]        [min]   [mAU*s]     [mAU]       %
----|-------|----|-------|----------|----------|--------|
   1  38.345 MM    2.1614 1.23776e4   95.44270  50.1498
   2  46.388 MM    2.9294 1.23037e4   70.00010  49.8502
```

Additional Info : Peak(s) manually integrated

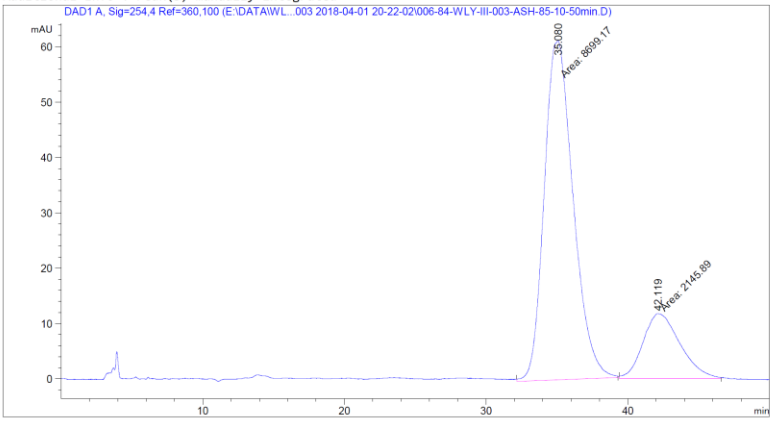


```
=====================================================================
                        Area Percent Report
=====================================================================

Sorted By             :      Signal
Multiplier            :      1.0000
Dilution              :      1.0000
Use Multiplier & Dilution Factor with ISTDs


Signal 1: DAD1 A, Sig=254,4 Ref=360,100

Peak RetTime Type  Width     Area      Height     Area
  #   [min]        [min]   [mAU*s]     [mAU]       %
----|-------|----|-------|----------|----------|--------|
   1  35.080 MM    2.3651 8699.16504   61.30151  80.2132
   2  42.119 MM    3.0356 2145.89233   11.78163  19.7868
```

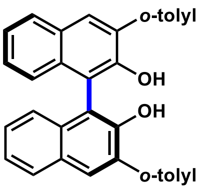

Additional Info : Peak(s) manually integrated

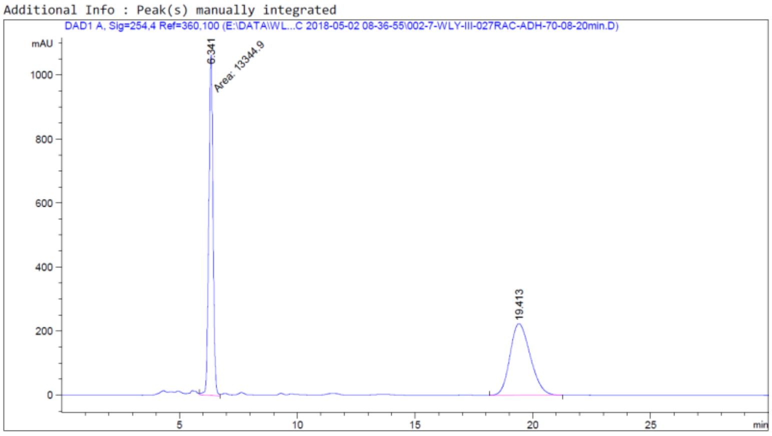

===
Area Percent Report
===

Sorted By : Signal
Multiplier : 1.0000
Dilution : 1.0000
Use Multiplier & Dilution Factor with ISTDs

Signal 1: DAD1 A, Sig=254,4 Ref=360,100

Peak #	RetTime [min]	Type	Width [min]	Area [mAU*s]	Height [mAU]	Area %
1	6.341	MM	0.2090	1.33449e4	1064.25916	49.9964
2	19.413	BB	0.7036	1.33469e4	223.53136	50.0036

Additional Info : Peak(s) manually integrated

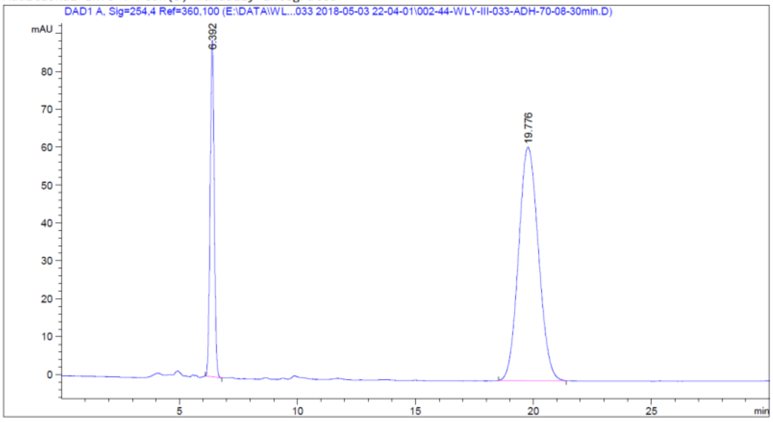

===
Area Percent Report
===

Sorted By : Signal
Multiplier : 1.0000
Dilution : 1.0000
Use Multiplier & Dilution Factor with ISTDs

Signal 1: DAD1 A, Sig=254,4 Ref=360,100

Peak #	RetTime [min]	Type	Width [min]	Area [mAU*s]	Height [mAU]	Area %
1	6.392	BB	0.1840	1069.56543	88.81834	22.9080
2	19.776	BB	0.6859	3599.39429	61.58450	77.0920

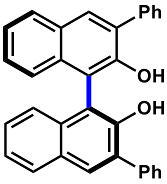

Additional Info : Peak(s) manually integrated

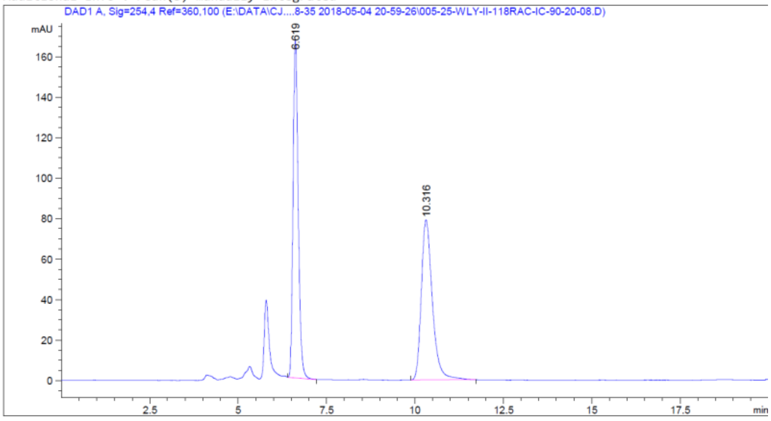

===
Area Percent Report
===

Sorted By : Signal
Multiplier : 1.0000
Dilution : 1.0000
Use Multiplier & Dilution Factor with ISTDs

Signal 1: DAD1 A, Sig=254,4 Ref=360,100

Peak #	RetTime [min]	Type	Width [min]	Area [mAU*s]	Height [mAU]	Area %
1	6.619	BB	0.1562	1695.65930	166.71983	49.7278
2	10.316	BB	0.3210	1714.22607	79.04620	50.2722

Additional Info : Peak(s) manually integrated

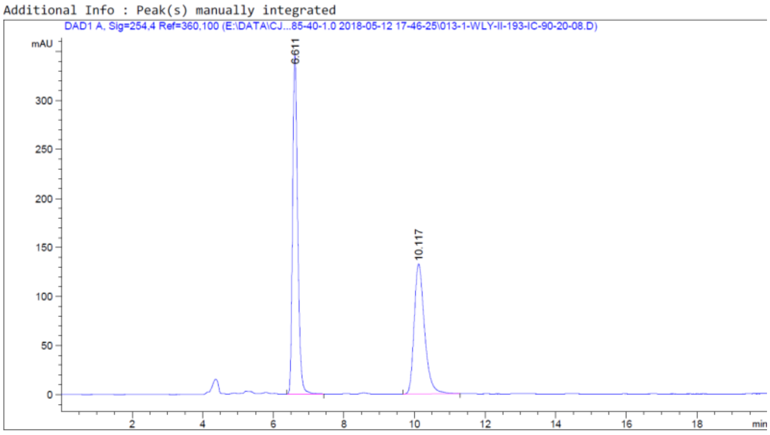

===
Area Percent Report
===

Sorted By : Signal
Multiplier : 1.0000
Dilution : 1.0000
Use Multiplier & Dilution Factor with ISTDs

Signal 1: DAD1 A, Sig=254,4 Ref=360,100

Peak #	RetTime [min]	Type	Width [min]	Area [mAU*s]	Height [mAU]	Area %
1	6.611	BB	0.1538	3463.72510	346.01978	56.1755
2	10.117	BB	0.3080	2702.16992	132.75162	43.8245

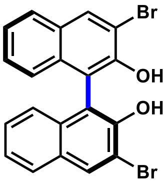

Additional Info : Peak(s) manually integrated

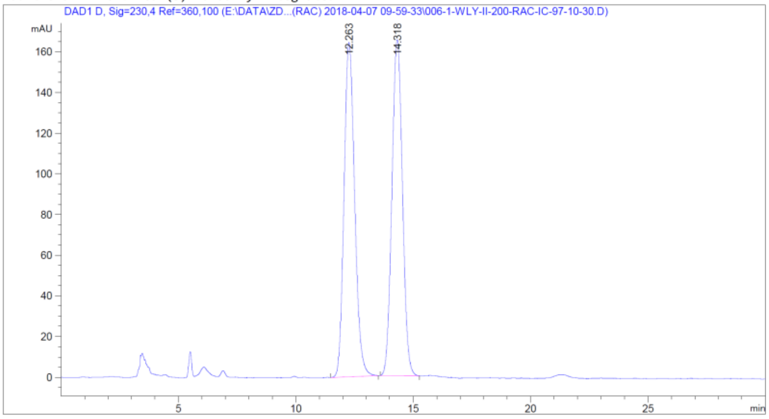

==

Area Percent Report

==

Sorted By : Signal
Multiplier : 1.0000
Dilution : 1.0000
Use Multiplier & Dilution Factor with ISTDs

Signal 1: DAD1 D, Sig=230,4 Ref=360,100

Peak #	RetTime [min]	Type	Width [min]	Area [mAU*s]	Height [mAU]	Area %
1	12.263	BB	0.4789	5155.60742	164.36372	50.0762
2	14.318	BB	0.4743	5139.91748	165.07063	49.9238

Additional Info : Peak(s) manually integrated

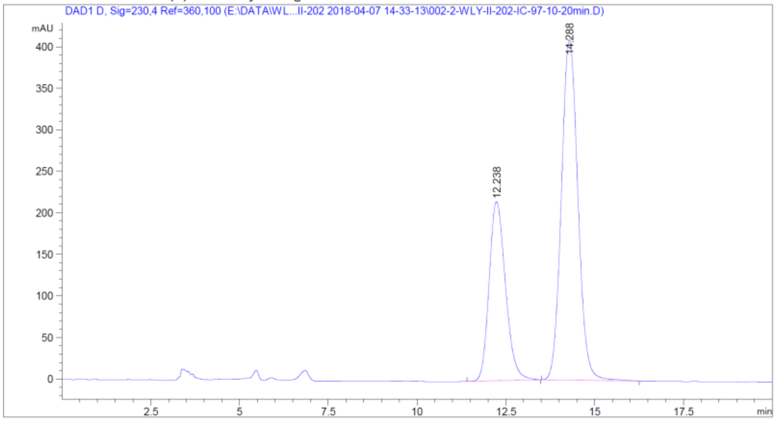

==

Area Percent Report

==

Sorted By : Signal
Multiplier : 1.0000
Dilution : 1.0000
Use Multiplier & Dilution Factor with ISTDs

Signal 1: DAD1 D, Sig=230,4 Ref=360,100

Peak #	RetTime [min]	Type	Width [min]	Area [mAU*s]	Height [mAU]	Area %
1	12.238	BB	0.4767	6913.74707	215.53433	34.2861
2	14.288	BB	0.4946	1.32511e4	409.40289	65.7139

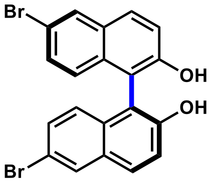

Additional Info : Peak(s) manually integrated

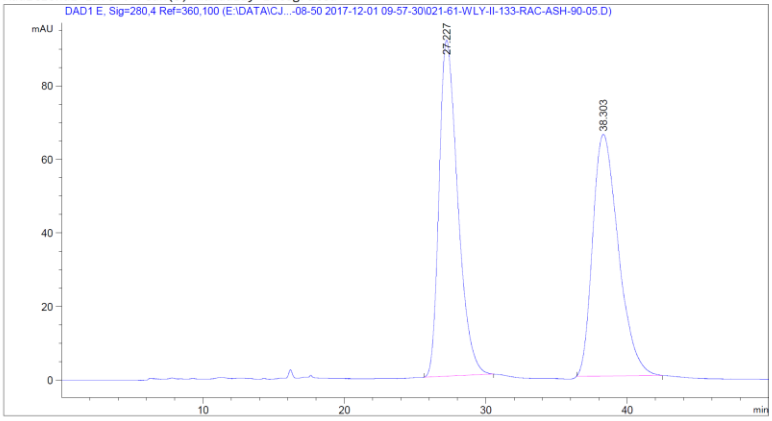

===
Area Percent Report
===

Sorted By : Signal
Multiplier : 1.0000
Dilution : 1.0000
Use Multiplier & Dilution Factor with ISTDs

Signal 1: DAD1 E, Sig=280,4 Ref=360,100

Peak #	RetTime [min]	Type	Width [min]	Area [mAU*s]	Height [mAU]	Area %
1	27.227	BB	1.0748	8392.92383	91.40069	50.2802
2	38.303	BB	1.4814	8299.37695	65.71684	49.7198

Additional Info : Peak(s) manually integrated

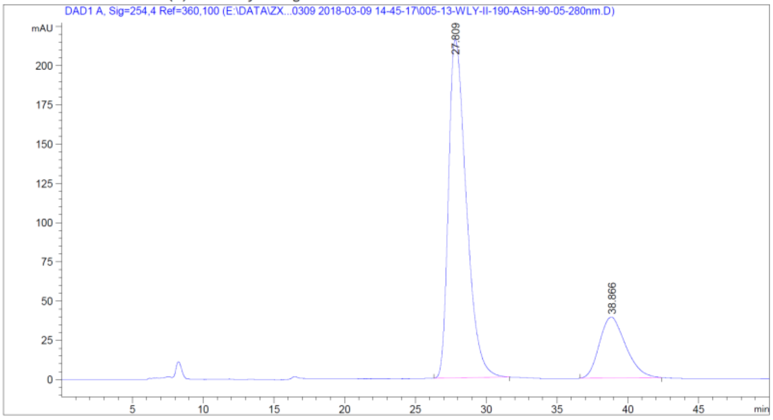

===
Area Percent Report
===

Sorted By : Signal
Multiplier : 1.0000
Dilution : 1.0000
Use Multiplier & Dilution Factor with ISTDs

Signal 1: DAD1 A, Sig=254,4 Ref=360,100

Peak #	RetTime [min]	Type	Width [min]	Area [mAU*s]	Height [mAU]	Area %
1	27.809	BB	1.0252	1.88863e4	215.54605	79.1298
2	38.866	BB	1.5017	4981.18848	38.80158	20.8702

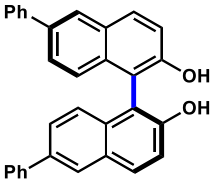

Additional Info : Peak(s) manually integrated

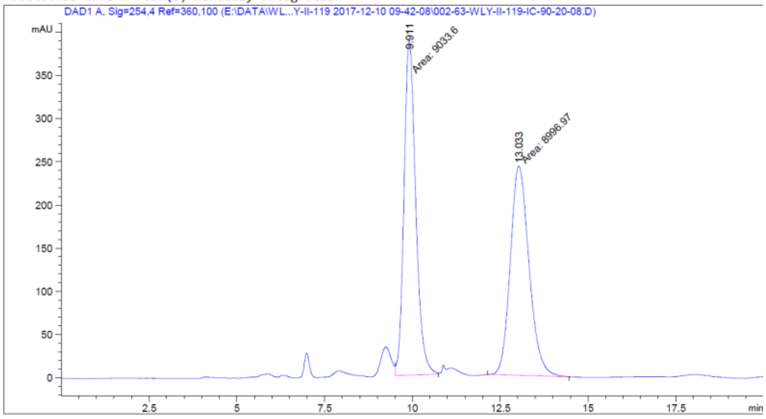

===

Area Percent Report

===

Sorted By : Signal
Multiplier : 1.0000
Dilution : 1.0000
Use Multiplier & Dilution Factor with ISTDs

Signal 1: DAD1 A, Sig=254,4 Ref=360,100

Peak #	RetTime [min]	Type	Width [min]	Area [mAU*s]	Height [mAU]	Area %
1	9.911	MM T	0.3882	9033.59766	387.84766	50.1016
2	13.033	MM T	0.6191	8996.97168	242.20374	49.8984

Additional Info : Peak(s) manually integrated

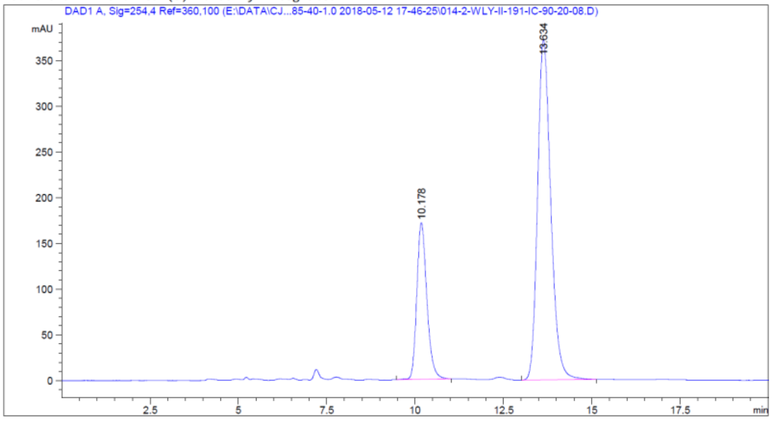

===

Area Percent Report

===

Sorted By : Signal
Multiplier : 1.0000
Dilution : 1.0000
Use Multiplier & Dilution Factor with ISTDs

Signal 1: DAD1 A, Sig=254,4 Ref=360,100

Peak #	RetTime [min]	Type	Width [min]	Area [mAU*s]	Height [mAU]	Area %
1	10.178	BB	0.3033	3404.15796	171.31363	26.0270
2	13.634	BB	0.3961	9675.18945	371.78561	73.9730

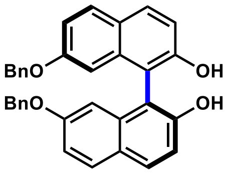

Additional Info : Peak(s) manually integrated

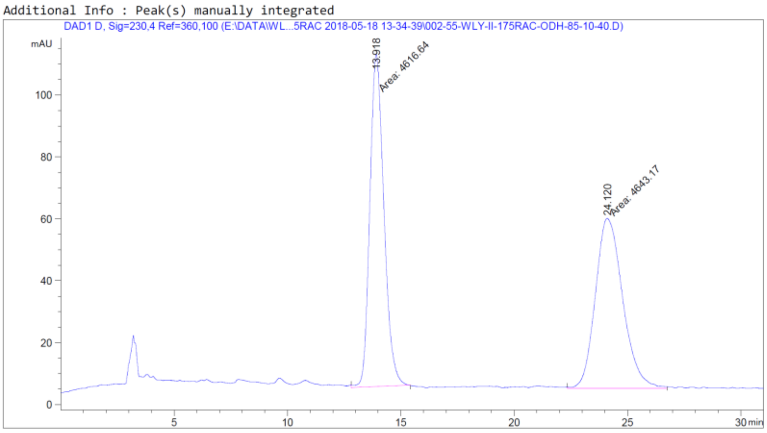

==

Area Percent Report

==

Sorted By : Signal
Multiplier : 1.0000
Dilution : 1.0000
Use Multiplier & Dilution Factor with ISTDs

Signal 1: DAD1 D, Sig=230,4 Ref=360,100

Peak #	RetTime [min]	Type	Width [min]	Area [mAU*s]	Height [mAU]	Area %
1	13.918	MM	0.7186	4616.64160	107.07887	49.8568
2	24.120	MM	1.4071	4643.16748	54.99638	50.1432

Additional Info : Peak(s) manually integrated

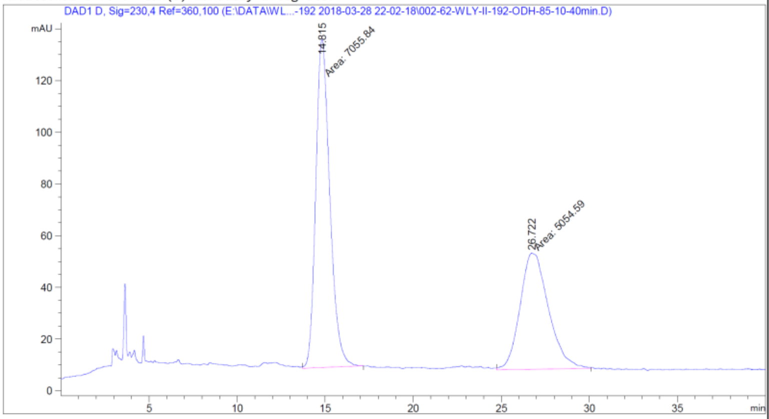

==

Area Percent Report

==

Sorted By : Signal
Multiplier : 1.0000
Dilution : 1.0000
Use Multiplier & Dilution Factor with ISTDs

Signal 1: DAD1 D, Sig=230,4 Ref=360,100

Peak #	RetTime [min]	Type	Width [min]	Area [mAU*s]	Height [mAU]	Area %
1	14.815	MM	0.9270	7055.84375	126.85791	58.2625
2	26.722	MM	1.8762	5054.59082	44.90165	41.7375

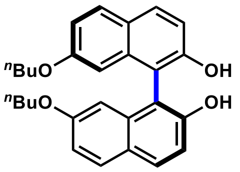

Additional Info : Peak(s) manually integrated

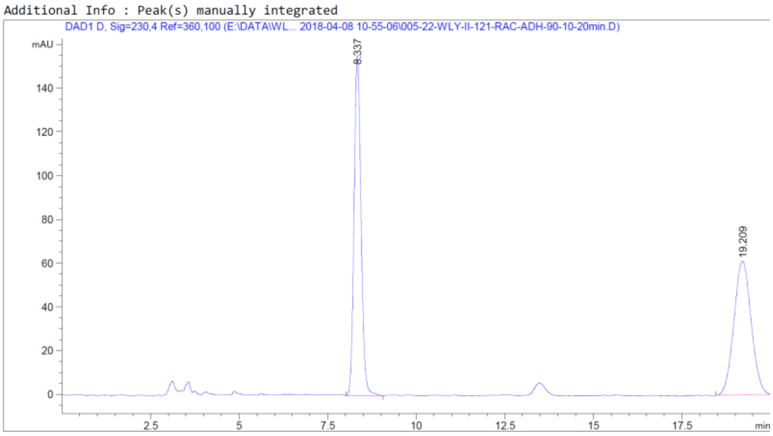

===
Area Percent Report
===

Sorted By : Signal
Multiplier : 1.0000
Dilution : 1.0000
Use Multiplier & Dilution Factor with ISTDs

Signal 1: DAD1 D, Sig=230,4 Ref=360,100

Peak #	RetTime [min]	Type	Width [min]	Area [mAU*s]	Height [mAU]	Area %
1	8.337	BB	0.2102	2129.50830	155.52692	50.7481
2	19.209	BBA	0.4113	2066.72485	61.15033	49.2519

Additional Info : Peak(s) manually integrated

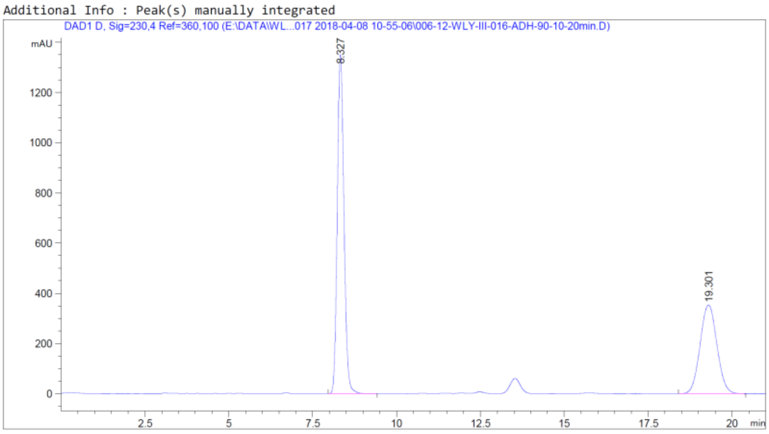

===
Area Percent Report
===

Sorted By : Signal
Multiplier : 1.0000
Dilution : 1.0000
Use Multiplier & Dilution Factor with ISTDs

Signal 1: DAD1 D, Sig=230,4 Ref=360,100

Peak #	RetTime [min]	Type	Width [min]	Area [mAU*s]	Height [mAU]	Area %
1	8.327	BB	0.2136	1.85824e4	1349.92126	60.1806
2	19.301	BB	0.5348	1.22953e4	353.02341	39.8194

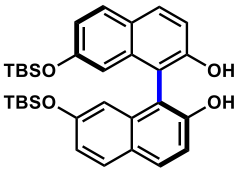

Additional Info : Peak(s) manually integrated

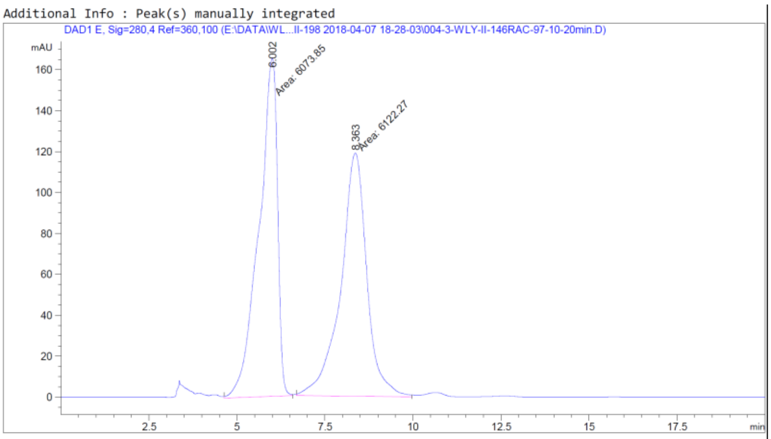

===

Area Percent Report

===

Sorted By : Signal
Multiplier : 1.0000
Dilution : 1.0000
Use Multiplier & Dilution Factor with ISTDs

Signal 1: DAD1 E, Sig=280,4 Ref=360,100

Peak #	RetTime [min]	Type	Width [min]	Area [mAU*s]	Height [mAU]	Area %
1	6.002	MM	0.6131	6073.85254	165.12057	49.8015
2	8.363	MM	0.8591	6122.27441	118.77021	50.1985

Additional Info : Peak(s) manually integrated

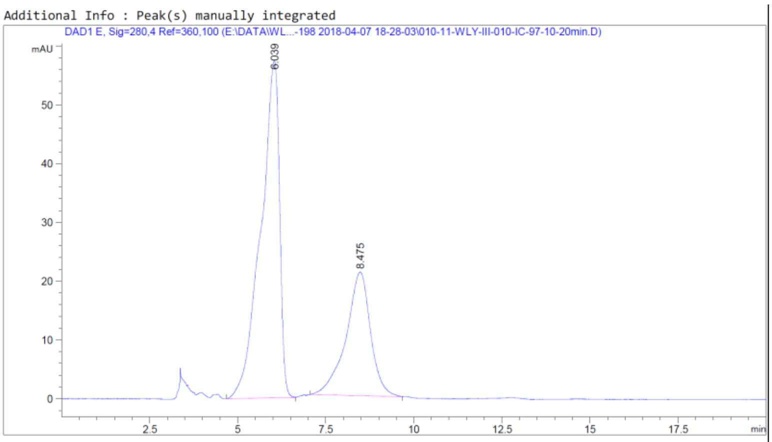

===

Area Percent Report

===

Sorted By : Signal
Multiplier : 1.0000
Dilution : 1.0000
Use Multiplier & Dilution Factor with ISTDs

Signal 1: DAD1 E, Sig=280,4 Ref=360,100

Peak #	RetTime [min]	Type	Width [min]	Area [mAU*s]	Height [mAU]	Area %
1	6.039	BB	0.4912	2114.36255	57.40987	67.5320
2	8.475	BB	0.5674	1016.54102	21.05134	32.4680

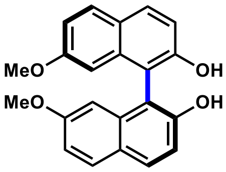

Additional Info : Peak(s) manually integrated

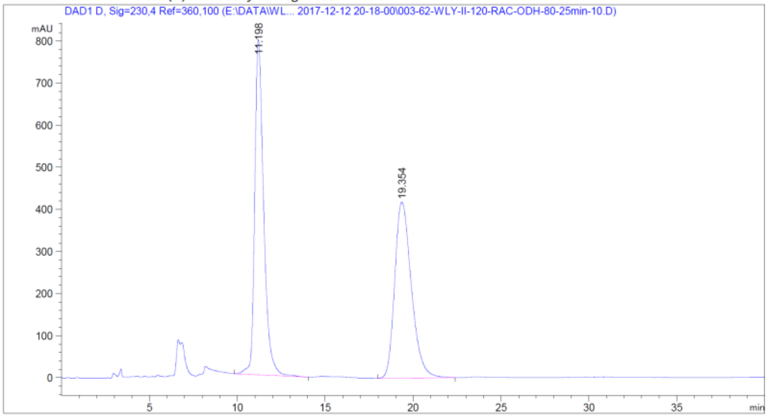

===

Area Percent Report

===

Sorted By : Signal
Multiplier : 1.0000
Dilution : 1.0000
Use Multiplier & Dilution Factor with ISTDs

Signal 1: DAD1 D, Sig=230,4 Ref=360,100

Peak #	RetTime [min]	Type	Width [min]	Area [mAU*s]	Height [mAU]	Area %
1	11.198	BB	0.5319	2.80968e4	795.80017	50.5371
2	19.354	BB	0.9584	2.74996e4	418.07074	49.4629

Additional Info : Peak(s) manually integrated

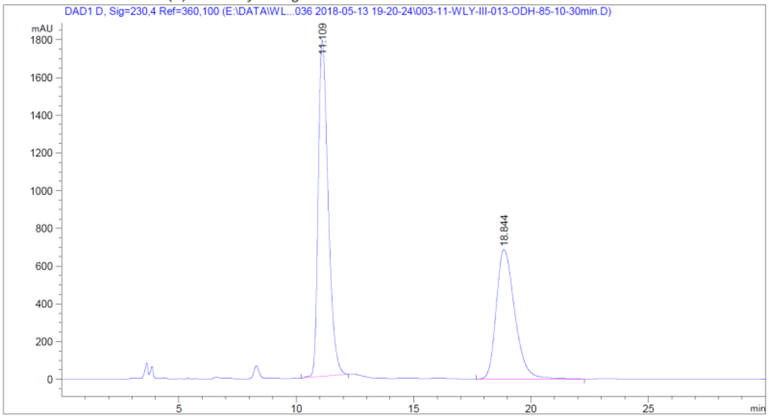

===

Area Percent Report

===

Sorted By : Signal
Multiplier : 1.0000
Dilution : 1.0000
Use Multiplier & Dilution Factor with ISTDs

Signal 1: DAD1 D, Sig=230,4 Ref=360,100

Peak #	RetTime [min]	Type	Width [min]	Area [mAU*s]	Height [mAU]	Area %
1	11.109	BB	0.4422	5.29766e4	1785.46069	58.5455
2	18.844	BB	0.7919	3.75114e4	687.09198	41.4545
